# Advanced non-fluoride approaches to dental enamel remineralization: The next level in enamel repair management

**DOI:** 10.1016/j.bbiosy.2021.100029

**Published:** 2021-10-29

**Authors:** Bernd Grohe, Silvia Mittler

**Affiliations:** a Lawson Health Research Institute, St. Joseph's Hospital, London, ON, N6A 4V2 Canada; b Department of Physics & Astronomy, University of Western Ontario, London, ON, N6A 3K7 Canada; c Department of Chemical and Biochemical Engineering, University of Western Ontario, London, ON, N6A 5B9 Canada

**Keywords:** Dental caries, Erosive tooth wear (ETW)/dental erosion (DE), (Non)-fluoride remineralization approaches, Protein/peptide mediated remineralization, Salivary and external calcium phosphate, Regenerative dentistry

## Abstract

In modern dentistry, a minimally invasive management of early caries lesions or early-stage erosive tooth wear (ETW) with synthetic remineralization systems has become indispensable. In addition to fluoride, which is still the non-plus-ultra in these early caries/ETW treatments, a number of new developments are in the test phase or have already been commercialized. Some of these systems claim that they are comparable or even superior to fluoride in terms of their ability to remineralize enamel. Besides, their use can help avoid some of the risks associated with fluoride and support treatments of patients with a high risk of caries. Two individual non-fluoride systems can be distinguished; intrinsic and extrinsic remineralization approaches. Intrinsic (protein/peptide) systems adsorb to hydroxyapatite crystals/organics located within enamel prisms and accumulate endogenous calcium and phosphate ions from saliva, which ultimately leads to the re-growth of enamel crystals. Extrinsic remineralization systems function on the basis of the external (non-saliva) supply of calcium and phosphate to the crystals to be re-grown. This article, following an introduction into enamel (re)mineralization and fluoride-assisted remineralization, discusses the requirements for non-fluoride remineralization systems, particularly their mechanisms and challenges, and summarizes the findings that underpin the most promising advances in enamel remineralization therapy.

## Highlights

• Non-fluoride approaches minimize harmful effects associated with fluoride compounds.
• Intrinsic approach: controlled remineralization via endogenous Ca-P-ions from saliva.
• Extrinsic approach: controlled remineralization via externally supplied Ca-P-ions.
• Most advanced system: extrinsic casein phosphoprotein - amorphous calcium phosphate.
• A promising intrinsic candidate: the P_11_–4 system, a self-assembling peptide.

## 1 Introduction

Dental diseases such as caries and the effects of erosive tooth wear (ETW; previous term: dental erosion; DE) are common and a major public health concern [1,2]. Both kind of events demineralize and soften the dental enamel, paving the way for further tooth decay and cavitation [1,2]. One goal of current dentistry is therefore to intervene early in the development of caries/ETW and to treat non-cavitated/submicrometer eroded enamel non-invasively through remineralization. For this purpose, fluoride is still the non-plus-ultra in enamel management. In the currently developing regenerative dentistry, however, new developments for targeted remineralization treatments are increasingly being used. If successful, they can prevent the progression of decay and improve or even restore the strength and function of the dental hard tissue [3]. In order to control the interventions knowledge of the molecular processes of enamel de- and remineralization is important.

Controlled by DNA, organisms use sophisticated mechanisms to mineralize organic-inorganic composites through the interaction of proteins (ultimately cells) with inorganic mineral phases. This takes place in an aqueous environment of dissolved organic and inorganic compounds in a cell compartment at a certain pH. Therefore, the properties of such formed biocomposites (e.g. teeth) result from the functionality of proteins that regulate biomineralization at the organic-inorganic interface, but also from the physicochemical environment in which these interactions occur [4,5].

Enamel mineralization is controlled by the formation of an isolated compartment within a cell aggregation (the tooth organ, also called enamel or dental organ) that can be found in a developing tooth [6,7]. This extracellular space is protected from external influences by two layers of cells, which ensure that the penetration of even the smallest ions is blocked and that the composition of the extra cellular space's electrolyte is only determined by the secretory and resorptive activities of these cells lining the space. Secretion of various components to the electrolyte of the ‘chamber’ (or matrix) must be constantly monitored, coordinated and varied in order to meet the constantly changing requirements of this multi-stage process [6]. Reith [8] suggests five stages of enamel formation: (I) secretion of an organic matrix into the cell compartment, (II) crystal nucleation and growth out of the organic matrix, (III) crystal elongation, (IV) removal of the organic matrix, and (V) crystal maturation. In its final state, mature enamel is acellular and highly mineralized. This means that there are no cells that supply the enamel with crystal growth medium in the event of, e.g., an acid attack [6]. Enamel is not able to remineralize itself; it needs to be remineralized by its environment [9,10]. It is, therefore, important to understand and to control the molecular mechanisms of enamel mineralization, particularly the remineralization processes, in order to preserve dental enamel.

Mature enamel is the hardest tissue in humans [11] and covers the entire crown of the tooth (supplemental material Fig. S1). It is made up of organic and inorganic components that are involved in its natural mineralization processes [12,13]. More precisely, dental enamel consists of tightly packed and intertwined mineral rods (also called prisms) that are bound by proteins, which belong to an interfacial layer system [2,14,15]. These rods or prisms in turn consist of protein-bound hydroxyapatite (HA) fibers that run parallel and at certain angles to the direction of the prisms (see Fig. S1) [2,14,16,17]. Enamel's composition and structure ensures a high degree of hardness and mechanical elasticity to avoid catastrophic failure [2].

Enamel is always in direct contact with a biofilm that is formed from saliva components (Fig. S2). The biofilm is composed of the acquired pellicle (bacteria-free) and the bacteria-containing plaque [18]. The acquired pellicle, an interfacial protein layer (up to 1 µm thick) between enamel and plaque, consists, e.g., of adsorbed glycoproteins, mucins, lipids [19,20]. Plaque in turn forms a transitional layer of adhered and aggregated bacteria with a bacterial density that decreases with increasing distance from the tooth surface (details are given in Fig. S2). At a sufficient distance, bacteria will spread to new areas [18]. Bacteria in direct contact with the pellicle are able to penetrate the protein layer [18]. The formation mechanism of the biofilm and its structure is largely determined by the adsorption of proteins and bacteria as well as their adherence and aggregation [18,21]. These processes are in turn based on interaction forces and entropic contributions (see Fig. S3) [21], [22], [23], [24].

Influenced by the biofilm, enamel mineralization processes usually take place when HA of enamel is dissolved (demineralization due to caries, or ETW) or when salivary proteins, calcium/phosphate ions, fluoride and other components remineralize locally dissolved enamel [2,13,25,26].

With regard to enamel degradation, a distinction is made primarily between caries [27,28] and ETW [2,29]. Diseases such as amelogenesis imperfect also lead to tooth decay, but with a completely different course of the disease [30]. Enamel demineralization by caries is caused by bacteria fermenting foods (carbohydrates) in dental plaque. This process creates organic acids (e.g., lactic, acetic, formic, propionic) that dissolve tooth mineral [25]. The acids readily diffuse in all directions, through the acquired pellicle (see Fig. S2) and into enamel. When they encounter acid-soluble mineral (HA), they begin slowly to dissolve it [27,28]. Caries lesion is now forming, which results in cavities over months or years (at pH values of 4.5 – 5) if the process is not stopped [11,27,31]. Erosive tooth wear (ETW), on the other hand, is induced by chemical degradation without the involvement of bacteria [2]. By definition, ETW is a combination of two or more of the following mechanisms: acid attack or erosion (loss of tooth structure caused by acids of diet (vinegar, apples, citrus fruits and sodas, containing, e.g., damaging citric acid and phosphoric acid) or recurrent vomiting [32,33,34] (Fig. S4)); attrition (mechanical loss by wear and tear of teeth caused by tooth-to-tooth contact [35]); abrasion (mechanical loss of tooth structure caused by contact of exogenous material that is forced over the surface of the enamel [36]); and abfraction (dynamic tooth wear caused by stresses due to flexure of a tooth under heavy lateral loads [37,38]). Contrary to caries, the acidic impact on dental enamel by ETW is characterized by a short-term process at a relatively low pH (values as low as pH ∼ 2 - 2.5) [2,29,39]. Typically, ETW starts with an acid attack, which softens and breaks down dental enamel. Enamel is therefore more susceptible to mechanical forces such as attrition or abrasion (see Fig. S5). If this cycle of softening/mechanical impact of the enamel is not broken, the tooth will decay or even be lost [2,40,41]. Recently, Bartlett et al. have proposed the Basic Erosive Wear Examination (BEWE), which ranks the extend of tooth wear, helping dentists in patient's risk management [42].

Enamel remineralization is the natural repair process for early caries lesions/early-stage ETW, in which – under the assistance of salivary proteins – calcium, phosphate and other ions are deposited into voids of demineralized enamel [3,13,25,26]. However, it is important to note that the remineralization process is not strongly promoted by just suppressing the demineralization process, or, in other words, that the suppression of demineralization "… is an essential function for promoting dental remineralization… ", as a recent publication suggests [43]. De-and remineralization are a complex combination of various processes running in parallel (crucial: the so-called 'central dark line' of enamel crystals). Remineralization involves capturing calcium (Ca) and phosphate (P) ions (sometimes fluoride) in slightly supersaturated saliva and directing these ions to carious and/or acid eroded sites where remineralization occurs. (for details please refer to the supplemental material, Section 3.2 and 3.3). At the same time, however, demineralization/degradation processes take place. Consequently, the rate of remineralization must be greater than the rate of demineralization in order to ultimately build up the enamel [13]. More details on enamel formation, its breakdown and remineralization (also on the nano-scale) are discussed in the supplement material.

The traditional fluoride-based treatment plays a major role in enamel repair, as it very effectively interrupts the demineralization/degradation process and supports the remineralization of the enamel (for details see Section 2) [25]. However, there are risk factors associated with too frequent fluoride treatments/intakes (e.g., fluorinated water, toothpaste etc.) or high fluoride concentrations that are not tailored to the patient. The former can lead to the development of dental fluorosis [44,45], the latter to a fluoride syndrome [46]. There is therefore a great need to develop remineralization systems that complement fluoride in its effectiveness or even replace it in the future.

In recent years, a better understanding of the physicochemical and biological mineralization mechanisms has influenced the development of a number of remineralization methods that go beyond fluoride-mediated remineralization [31]. These approaches are particularly helpful when developing new treatment options for people with a high risk of caries or ETW [31]. We will therefore review individual non-fluoride remineralization systems that are currently emerging in test phases or are already on the market. We have divided these systems into so-called intrinsic (endogenous) and extrinsic (exogenous) systems. In the group of intrinsic systems there are e.g. proteins, peptides, dendrimers, which are able to adsorb to specific sites (to be remineralized) and to accumulate the calcium and phosphate present in the saliva. The ion accumulation leads to a supersaturation of calcium and phosphate and ultimately to the remineralization of enamel [23,31]. This group is referred to in the following as the ‘intrinsic (endogenous) calcium phosphate approach ' (iCP). In the group of extrinsic systems there are, e.g., nanocrystalline and amorphous calcium phosphates, which are provided to the sites to be remineralized via an external supply [3]. The supplied calcium phosphate solids are either dissolved and remineralize local prism HA, or they adsorb to enamel prisms and fuse with the prism crystals [47,48]. Since calcium and phosphate are supplied from external sources, this group is referred to below as the ‘extrinsic (exogenous) calcium phosphate approach’ (eCP). Note: If desired, fluoride and other compounds can be added to both types of approaches, the iCP and the eCP, depending on, e.g., the patient's treatment needs.

The characteristics of caries and ETW presented above are intended to provide an overview of possible influencing factors on these clinical pictures. However, it should be emphasized at this point that the non-fluoride approaches discussed in the present review are only therapeutic options for early caries lesions and early-stage ETW [31,33]. As with fluoride use, for successful treatment, caries can only be non-cavitated and ETW-damaged enamel can only show acid exposure but no signs of mechanical attack (submicron impacts are treatable). For ETW these treatment approaches can also be used as one tool among many to counteract the progression of ETW (besides e.g. avoid acid and mechanical influences, eat calcium and phosphate-rich foods such as cheese etc. [33,35,39]).

Following a brief chapter on traditional fluoride remineralization, the article discusses the requirements for non-fluoride remineralization systems, particularly their mechanisms and challenges, and reports on the findings that underpin the most promising advances in enamel remineralization therapy for early caries lesions and early-stage ETW.

## 2 Effects of traditionally used fluorides on enamel remineralization processes

Fluoride has a very beneficial effect on the mineralization process of dental enamel. Fluoride helps in two ways: it interrupts the demineralization process very effectively and supports the remineralization of enamel. These properties of fluorides are still used in some of the non-fluoride approaches by adding fluoride to them, for example to increase the hardness of the remineralized enamel. For this reason, fluoride affected mineralization processes are briefly discussed here.

In the event of an acid attack, in which the pH value drops to values between 4.5 and 5.5, HA dissolves and - in the presence of fluoride - fluorapatite (FA; Ca_5_(PO_4_,CO_3_)_3_(F)) begins to form [49]. For this reason and because the solubility of FA is lower than that of HA [50], the enamel dissolution process slows down. The calcium and phosphorus in enamel that is continuously lost during the de-remineralization cycles is recovered as FA and thus constantly increases the FA/HA ratio in dental enamel [13,51,52]. However, optimized fluoride concentrations are key to the formation of FA, because fluoride concentrations of > 5 ppm lead to the formation of calcium fluoride and to uncontrolled high reaction rates. In contrast, concentrations of ∼ 1 ppm fluoride (at physiological pH) strongly promoted the formation of the FA phase, at a rate about twice as high as in the absence of fluoride ions [53], [54], [55]. Optimal conditions for remineralization or, conversely, a significant inhibition of demineralization, was found with increasing fluoride concentrations from 0.2 to 2.0 ppm for a pH decrease from 5 to 4 [56]. Margolis et al. determined a "protection" against the demineralization of apatite in the presence of 0.025 ppm fluoride (physiological Ca and P concentrations; pH 4.3) [57].

Another aspect of FA formation is the rate of reaction and the phases formed. Apart from a possible formation of calcium fluorides (fluoride > 5 ppm), fluorapatite can form via too rapid mineralization rates if the fluoride concentration is ∼ 2 - 5 ppm (pH-dependent) [53,58]. Deposits similar to those observed for too fast HA reactions are the result (see supplemental material, Section 3.2.). Theses outcomes have been confirmed by García-Godoy & Hicks [59], who have reported high mineralization rates and surface enamel pore obturation, a process that resulted in disruption of Ca, P, and F diffusion to the underlying demineralized lesion. The latter also suggests that diffusion through the pores is reaction rate determining [13]. However, it was initially not clear whether the solids formed are really mineralized or an amorphous precursor phase [53]. Nancollas and coworkers have found inhibited crystal growth when 0.05 – 2 ppm fluoride was present and, more importantly, a delay of crystallization in the presence of 1 ppm fluoride [60,61]. But, instead of promoting an amorphous precursor phase, it is thought that the inhibitory effect of fluoride on apatite formation is due to a mismatch of the fluorapatite crystals formed on the hydroxyapatite lattice [60,61]. This implies that only a small proportion of the lattice spaces of the hydroxyapatite lattice are occupied by fluoride [58]. Moreno et al. [62] go one step further and have postulated that in the presence of fluoride ions, the percentage of lattice surfaces involved in crystal growth is increased due to the greater thermodynamic driving force for precipitation. This means that not only a part of the fluoride lattice occupation, but the entire crystal contributes to the hindered reaction [58].

**Fig. 1:**
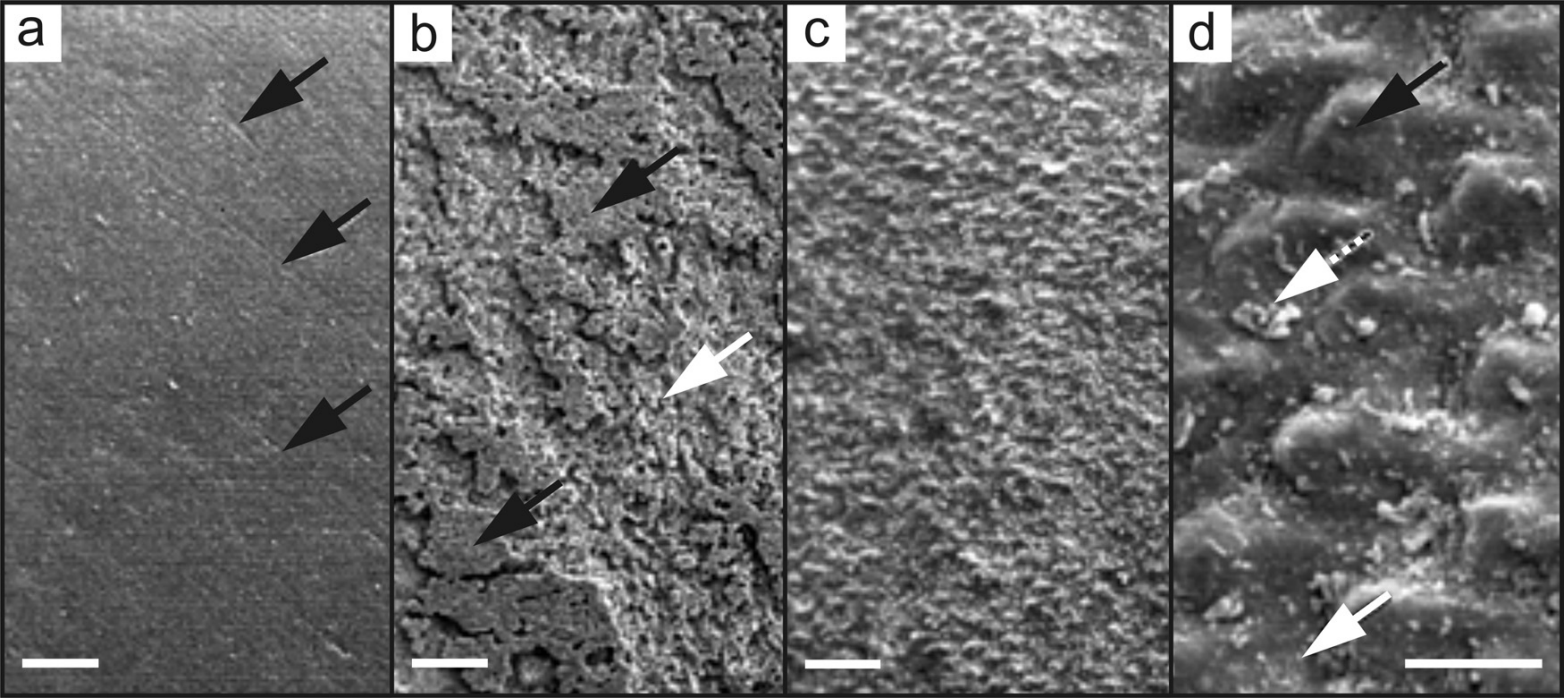
Scanning electron micrographs (SEM) of dental enamel surfaces on cut blocks from primary lower molars (starting material: caries-free; no other gross surface defects). Images were taken a) before enamel surface treatment. The surface is smooth, presenting some scratches caused by the person's tooth-brushing habits (black arrows); b) after etching with 37% phosphoric acid gel (Dentsply - Caulk) for 30 s (black arrows: original surface, white arrow: new exposed enamel); c) after tooth-brushing the acid treated enamel surface with a fluoridated dentifrice; d) from a magnified section of c), the rods (black arrow), the interrod area (white arrow) and some remnants (insoluble abrasive component of the dentifrice, dotted white arrow) are well recognizable. All images are taken from [15] and slightly modified (copyrighted and licensed work under Creative Commons CC BY-NC 4.0). Scale bars in a,b,c: 20 µm; in d: 5 µm.

The controlled use of fluoride not only adjusts the diffusion of Ca and P precisely to the areas to be remineralized and tailors the reaction rate; the product, the (re)mineralized fluorapatite, is also about 3–5 times harder than hydroxyapatite [63,64] and less soluble [50]. It is important to note that all of these outcomes are achieved at pH values of ∼ 4 - 5, which allows for moderate acidity in the oral cavity [52]. Acid attacks caused by bacteria or sucrose are therefore better averted. Hence, to decrease the severity of the destruction to the demineralized tissue, dental fluoride treatment is very widespread today. In addition, in some countries fluorides are added to drinking water [13,65]. On the other hand, excessive fluoride concentrations should be avoided as it increases the risk of developing dental fluorosis in children (recognizable by brown and mottled teeth, caused by the underdeveloped formation of the enamel) [44,45] or occult (hidden) caries lesion, the so-called fluoride syndrome (found across all age groups). The latter is due to excessive remineralization of the enamel surface, which prevents Ca, P and F from reaching the subsurface regions of the lesions (i.e. no remineralization of the subsurface lesions) [46]. An experimentally initiated process of enamel de- and remineralization (fluoride present) is shown in Fig. 1.

Another factor that influences the in-vivo mineralization is the high concentration of, e.g., (bi)carbonate, sodium, pyrophosphate, (glyco)proteins, peptides and related compounds [13]. These ions and macromolecules can hinder the apatite remineralization process by decreasing the supply of Ca and P ions to the site of remineralization (by diffusion limitation and chelating) [58]. Such sites are found within enamel, where crystals are partially covered with proteinaceous material and, therefore, only part of the available surface is remineralized (organic material could possibly serve as a membrane) [58,66]. These sites (places of high organic content) can be found in lesions as well, where mineralization rates may be two orders of magnitude slower than the rates found in-vitro [67]. Of course, in this context the biofilm (consisting of the pellicle and plaque) must be taken into account that forms the interface between enamel and saliva (see Fig. S2 and S3). These organic layers may either act as a diffusion membrane or a reservoir for mineral constituents [13].

## 3 Effects of non-fluoride approaches on enamel remineralization processes

Under healthy conditions, saliva is thermodynamically an effective remineralization medium for surface enamel/lesion. However, subsurface lesions remineralize relatively slow as the diffusible calcium and phosphate concentrations are low. Therefore, there are distinct advantages by developing a mineralization strategy that pursues both the prevention of demineralization and the promotion of remineralization [68]. As mentioned above, enamel treatments with fluoride are a step in this direction. Instead of the hydroxide ion, fluoride is incorporated into the apatite. This makes the mineral phase less soluble and harder, but also the remineralization process more controllable. However, the fluoride treatments are topical and mostly result in FA, which merely coats surface enamel [67]. In addition, although effective in enamel mineralization, fluorides do not have the potential to promote the growth of apatite crystals [68,69]. Since this has now been understood, there has recently been a general shift in research efforts from reparative to regenerative therapies aimed at replacing diseased tooth tissue with tissue that is more biologically similar [70].

Since mature enamel is acellular and does not resorb or remodel by itself, efforts in tissues engineering led to remineralization systems that show a strong potential for the regeneration of the enamel microstructure. These systems, Philips referred to them as biomimetic systems or the calcium phosphate systems [31], should ideally combine the following characteristics: a) ensuring an increased Ca and P concentration directly at the sites to be remineralized (which increases the remineralization rate) and b) the guarantee of sufficient fluoridation and/or presence of strongly adsorbing macromolecules (e.g., peptides that hinder the dissolution of enamel) to prevent demineralization [25,26,71].

The remineralization systems currently under development are based on the proteins amelogenin and dentine phosphoprotein or on synthetic peptides that are very similar to these salivary proteins and their physiological properties. Very recently statherin peptides joined this group. All of these systems adsorb specifically to the sites to be remineralized and accumulate Ca and P ions from saliva, which leads to a local supersaturation of these ions and ultimately to the crystallization of HA [23,31]. Since the Ca and P ions available for remineralization are enriched endogenous from saliva, the process can be described as intrinsic. Philip [31] describes these systems as "non-fluoride enamel remineralization systems’, which is no longer quite true today, since fluorides can be added to these systems (see below for details). In contrast, calcium phosphate remineralization systems, such as nano-hydroxyapatite particles, peptide or silicate stabilized calcium phosphate phases, and polyphosphate systems, function on the basis of their external Ca and P supply to the places to be remineralized (via, e.g., tooth paste) [3,31]. Thereby, the supplied calcium phosphates are either dissolved and remineralized locally as HA or FA, or calcium phosphate particles are incorporated into, e.g., voids of enamel prisms, which then fuse together with the prism crystals [47,48,72]. Since Ca and P ions are added to saliva, these calcium phosphate approaches can be referred to as extrinsic (exogenous). Philip [31] describes these systems as "fluoride boosters”, although currently some of these systems show excellent results even without the fluoride additives (see below for details). In the following, all remineralization systems based on an ‘intrinsic (endogenous) calcium phosphate approach’ are referred to as iCP and all systems based on an ‘extrinsic (exogenous) calcium phosphate approach’ are referred to as eCP. Note: if desired, other compounds can be added to all of these approaches. It should also be mentioned that antibacterial drugs (possibly histatin 3 peptides) can be administered with almost any of these systems [73,74]. A number of these remineralization strategies are already in the development phase, especially those from the group of the eCP systems. A few of them are in the clinical phase; and they can be used as a treatment for early caries lesions as well as early-stage (submicrometer) ETW (see Section 4). However, a handful of the described systems cannot be used under strict clinical conditions, because they have not yet been properly tested, have side effects, or have controversial therapeutic efficacy. In the following the most promising strategies are summarized.

### 3.1 Intrinsic (endogenous) calcium phosphate approaches (iCP)

The most potential iCP systems include the DSS peptides derived from dentine phosphoprotein (DPP), the amelogenin-based polypeptides and derivates (e.g., leucine-rich amelogenin peptides, enamel matrix (amelogenin) derivative), the so-called P_11_–4 peptide (amelogenin inspired) and poly (amido amine) dendrimers [31,71]. Unfortunately, strategies based on the protein statherin have rarely been described which could be due to the fact that other systems are currently more promising. The only iCP strategy being clinically tested at the moment is the P_11_–4 peptide-assisted remineralization.

**Dentine phosphoprotein (DPP)-derived peptides.** Among these peptides, those that contain eight-fold repeats of aspartate‑serine‑serine (8DSS) are the most active in promoting biomineralization [75]. 8DSS peptides not only strongly bind free Ca^2+^and PO_4_^3–^ ions in saliva, they also adsorb to the HA surface and form strong bonds with them [75,76]. 8DSS peptides thus meet two conditions: a) they can block the demineralization process and the dissolution of Ca^2+^and PO_4_^3–^ ions in the surrounding medium (due to their adsorption to enamel crystals) and b) they promote the capture of these ions from saliva in order to initiate and drive the mineralization process. Recently, Zheng et al. 2019 [77] implemented an in-vivo rat caries model study and found that 8DSS is a promising remineralization agent that exhibits comparable effects to NaF (*p* < 0.05). However, 8DSS peptides have mostly been investigated in-vitro, with the one exception mentioned above. It is therefore not known, for example, whether its strong binding to Ca leads to a CaP built-up in-vivo and thus to tartar formation. Future in-vivo studies need to clarify this possible disadvantage. Nevertheless, the 8DSS strategy holds great promise as a non-fluoride biomineralizing agent [78].

**Amelogenin-based polypeptides and derivates.** The use of amelogenin for remineralization treatment of early caries lesions and early-stage ETW is obvious (for details see the excellent review by Ruan & Moradian-Oldak [79]). In fact, studies of amelogenin's (re)mineralization ability were carried out using the protein, e.g., in solution or in agar gels. In solution, amelogenin promoted the oriented bundle formation of rod-like FA in a dose dependent manner [80], in agar gel the amelogenin-supported enamel mineralization increased the surface micro-hardness significantly [81]. However, clinical studies are still pending. One reason could be the costly processing (purification, etc.) of the full-length protein. Therefore, the relationship between functional domains of amelogenin and the growth of enamel crystals may have sparked great interest in the development of amelogenin-based polypeptides. Particularly noteworthy is the leucine-rich amelogenin peptide (LRAP). Unlike amelogenin, the LRAP is non-phosphorylated. It consists of 56 amino acids, the first 33 amino acids starting from the N-terminus and the last 26 amino acids starting from the C-terminus of the parent amelogenin protein [82] (these domains have been reported to guide crystal growth of HA [83]). In-vitro studies have shown that the leucine-rich amelogenin peptide can reduce the depth of the lesions and promote the linear growth of mature enamel crystals along the c-axis, but also block demineralization processes [80,[84], [85], [86], [87]]. More importantly, the LRAP was able to improve the remineralization of surface lesions on extracted teeth in-vitro by promoting both the nucleation and growth of enamel crystals. Here, LRAP was even more effective than the parent full-length amelogenin [84], [85], [86], [87], [88], [89]. In addition, a host-guest hydrogel system with the peptide encapsulated in chitosan led to the formation of a dense apatite layer on the defective surface [85]. A recent clinical study demonstrates that such a system promotes the remineralization of early caries lesions and can inhibit *Streptococcus mutans* biofilm formation, lactic acid production and metabolic activity over a prolonged period of time [90].

Revealing the potential of the enamel matrix derivative Emdogain® (EMD) for periodontal tissue regeneration in clinical settings [91], [92], [93], [94], Cao et al. [95] encouraged EMD to be used to correct enamel defects. The use of the derivative in-vitro studies, in combination with a chloride-based agarose hydrogel, resulted in the successful formation of enamel prism-like structures (Ca/P ∼ 1.69, similar to the ratio in HA).

A drawback of amelogenin or its peptides/derivates is that their mediated enamel remineralization processes take an extended amount of time, making them - if there is no modification-breakthrough in the future - unsuitable for clinical use [79,96]. In addition, there is as yet no evidence that in-vivo studies show similar results than in-vitro studies demonstrating amelogenin-mediated biomineralization [79].

**Poly(Amido Amine) dendrimers (PAMAM).** PAMAM are able to mimic the formation and function of organic matrices in modulating the mineralization processes of enamel. These so-called “artificial proteins” can be modified with a range of reactive functional end groups to act as analogous of amelogenin [97]. Chen et al. [98] reported that these highly branched polymers are characterized by internal voids and a defined size. PAMAM tailored in this manner has recently shown in-vitro studies that amphiphilic, carboxyl-terminated, and phosphate-terminated dendrimers exhibited a strong tendency to self-assemble and form organic templates [98], [99], [100], [101], [102]. The crystals formed by these organic templates had the same structure and orientation as the intact enamel, with the HA rods closely paralleling the original prisms [98]. Using phosphate-terminated PAMAM in-vitro created a new HA layer on acid-etched enamel with HA rods that were up to ∼ 11 µm in length and adapted in their orientation to the natural enamel [99]. By conjugating alendronate to PAMAM, the dendrimers created crystal layers with a 95.5% hardness recovery in-vitro, and a significant enamel remineralization in the oral cavity of rats [101].

However, although PAMAMs have the potential to substitute amelogenin as synthetic analogues for biomineralization (overcoming efforts associated with extracting, purifying etc. of the natural protein), they are still far from clinical trials. Moreover, PAMAM-mediated enamel remineralization is time-consuming, and, unless the process cannot be accelerated, it is not practical for clinical application [31]. In addition, it is not clear whether these compounds are able to prevent demineralization processes. A solution to the latter problem could be the conjugation of a statherin peptide (N-terminal DDDEEK-C; **p**S**p**S was substituted by DD) to PAMAM, a combination that strongly interacts with HA due to the peptide. Gou et al. [103] has successfully tested the combination with regard to adsorption to HA, but unfortunately not yet its ability to prevent demineralization and promote remineralization.

Worth mentioning is a recently reported PAMAM application strategy, capable of combining anti-bacterial and mineralization properties. Synthesizing a phosphorylated version of PAMAM by means of a one-step method, an antibacterial drug (i.e. apigenin) was loaded inside the hydrophobic cavities of the phosphorylated PAMAM dendrimer [104]. When carrying out in-situ experiments, PAMAM could bind to dentin and effectively occlude the open dentin tubules. In the meantime, the released apigenin was able to eradicate the acid-producing bacteria (*Streptococcus mutans*) [104].

**Statherin peptides and composites.** Using statherin and its derivates for remineralization processes is at the very beginning. Systems or methods are not developed yet. However, it is worthwhile to have a look at some studies and advances they show. Statherin (human; ∼ 5 kDa; 43 amino acids) plays an important role in preventing the excess of CaP precipitation on surfaces (enamel and dentin) and in the salivary fluids by inhibiting CaP crystallization and capturing Ca and P ions [26,[105], [106], [107]]. The N-terminus of statherin is highly acidic, vital in electrostatically interacting (∼ 1/3 of the molecule) with calcium of CaP (e.g. enamel surfaces), and is the crucial part of the protein for remineralization processes [107,108]. When adsorbed, the first 12 amino acids (sequence 1–12; SN12: DSpSpEEKFLRR IG) are in close proximity to the crystal surface and form an α-helical structure upon adsorption [107,108]. By revealing the function of statherin, it was found that within the N-terminal fragment SN15 (DSpSpEEKFLRR IGRFG) apparently only the first 6 - 10 amino acids interact strongly with enamel, while for the amino acids 6–15 a function is suggested that leads to the capture of Ca and P [107], [108], [109]. To prevent demineralization, this domain (SN9) was recently 'rediscovered' [110].

Based on these findings, several laboratories have recently started to examine statherin sequences (especially in the SN15 range) for their suitability for (re)mineralizing dental enamel. One of the first steps in this direction was the synthesis of a dephosphorylated SN_D_15 (DDDEEKFLRR IGRFG; serine replaced by aspartic acid) attached to elastin-like recombinamers (ELR) and its use to control the synthesis of complex nanostructures [111,112]. The work of Li et al. [113], who used the SN_D_15-ELR recombinamers to coat titanium surfaces, is one step further. Treating the coated Ti discs in Ca and P ion containing media resulted in the formation of an amorphous calcium phosphate (ACP) layer. Similarly, using membranes, Elsharkawys et al. [114] crystallized spherulite-like (fluoride) apatite structures via disorder-order interactions, induced by SN_D_15- ELR. The study demonstrates that SN_D_15-ELR is quite effective in capturing Ca and P ions. A recent study, dealing with enamel remineralization in more detail, used SN_D_15-ELR to mineralize fluorapatite (FA) in a fluoridated calcium phosphate solution [115]. The process forms either a layered structure of ordered FA on SN_D_15-ELR-coated borosilicate glass or a needle-like FA crystal structure on borosilicate glass, with SN_D_15- ELR in solution. Another approach was chosen by Yang et al. [116] who fabricated a biofilm‐based material containing DDDEEK (SN_D_6). While the biofilm strongly binds to metal the SN_D_6 sequence is used to capture Ca and P ions and to mineralize HA. In this way, Ti_6_Al_4_V screws for implants were coated with biofilm in order to mineralize HA layers on the metal surfaces. Siqueira and co-workers recently went in a slightly different direction. In an attempt to control the mineralization rates of CaP, they used the principle of "more is stronger" (as previously shown for osteopontin peptides [117]) and synthesized a double SN9 (SN9-SN9) peptide. As expected, this peptide inhibited CaP formation stronger than the individual SN9, but did not promote remineralization as they falsely and misleadingly claim [43].

Although these studies do not yet show any work on the remineralization of enamel (or HA or another CaP), they demonstrate worthwhile approaches to tools based on statherin (such as, e.g., SM_D_15 or SM_D_6) and methods that, when perfected, can be developed into beneficial remineralization systems. For example, it was found that both the SN_D_15-ELR and the SN_D_6 biofilm coatings act as a nucleator for CaP crystallization and ultimately as a promoter for the formation of crystalline CaP films (see above).

These findings were used by Liu et al. [118], who synthesized an additional cysteine to the DDDEEK peptide (DDDEEK-C, SN_D_6-C) and investigated the remineralization capability of the peptide on (a) extracted human teeth and (b) in-vivo in rats. For the experiments with (a) human teeth, the enamel of molars was first etched in phosphoric acid (37%) and subsequently treated (twice a day for 8 days) either with SN_D_6-C, with NaF (positive control) or with deionized water (negative control), before immersing them in artificial saliva at 37 °C. For the experiments (b) with the rates, healthy rats were fed a cariogenic diet and infected with bacteria. The rats were then treated twice a day for 4 weeks with SN_D_6-C, with NaF or with deionized water. Both the human molars and the teeth of the rats showed pronounced remineralization when treated with SN_D_6-C or NaF. In addition, the remineralization potential of SN_D_6-C appears to be stronger than that of NaF. The study also shows that DDDEEK-C has a dual function: it adsorbs to enamel and induces remineralization. However, it is not yet clear which functions the individual amino acids have in these processes. Similarly, Yang et al. [119] recently demineralized dental enamel (phosphoric acid, 37%), but used the phosphorylated peptide version DpSpSEEK-C (SN6-C) for remineralization. In addition, NaF (positive control) and deionized water (negative control) was used. After immersing the enamel samples in artificial saliva (37 °C) for two weeks, the enamel treated with peptide showed pronounced remineralization and an obviously improved micro hardness compared to the negative control. However, the micro hardness did not match the hardness of the original enamel sample. Wang et al. [120] used the phosphorylated version as well, but synthesized five additional glutamic acid units onto the peptide. This peptide (DpSpSEEK-EEEEE [SN6-EEEEE]) cannot only induce remineralization it also shows a remineralization rate that is significantly higher than the rate induced by DpSpSEEK-C peptides. It is assumed that the E_5_ modification is responsible for this rate enhancement [120]. It is also believed that the long E_5_ tail adsorbs on the enamel surface, while the SN6 acts as a Ca and P ion "catcher" [120]. However, previous work on peptide adsorption has shown that phosphate groups interact more strongly with HA than carboxylates [121]. In general, this effect of promoting mineralization (and slowing demineralization) in the presence of increasingly acidic compounds (but decreasing compound concentration) is not new and was, to the best of our knowledge, first reported by Clark et al. [122] for protein crystal interactions and later by Elhadj et al. [123] who examined the peptide-controlled crystal growth at very low concentrations via AFM [123].

Comparative studies on the adsorption of SN6-EEEEE, SN_D_6-C and SN6-C are a necessary task for the future. In addition, peptide concentrations need to be optimized in order to determine the optimal adsorbed number density, which in turn influences the quality of enamel remineralization. Besides, it is not yet clear whether a statherin peptide treatment provides superior remineralization results compared to a fluoride treatment. These and, of course, a number of experiments are still missing that take the biological environment of in-vivo remineralization into account. Finally, clinical studies and trials are necessary to test the quality of the remineralization of caries lesions (especially the subsurface lesions) and the biocompatibility. For example, in clinical studies, N-terminal peptides in the range of SN6 - SN15 (or their dephosphorylated versions) could be delivered to caries lesions or acid-attacked teeth via a varnish/mouthwash. To increase the anti-bacterial potency of such treatments, peptides of, e.g., histatin 3 might be added. In addition, the long-term effect of the statherin peptides on the condition and health of enamel (also in comparison to other remineralization systems) and the question of whether the statherin peptides can provide superior remineralization (especially over fluorides) should be an important part of future tests and clinical trials.

Finally, the progress made in recent years in the development of peptide sequencing and production shows that statherin peptides are perfectly suited to act as a remineralization mediator for amorphous calcium phosphate (ACP) alongside casein. Tailoring SN3 - SN9 sequences (perhaps non-phosphorylated), and even a duplicate, a SN9-SN9, could serve as a remineralization agent in conjunction with ACP. This was already considered by Cochran et al. [3] and it should be thought through again. Another example of its use are combinations of statherin peptides with natural products. Recently, a SN_D_6-C - tannic acid conjugate was immersed in artificial saliva and adsorbed to demineralized (phosphoric acid, 37%) dental enamel disks (via SN_D_6-C). It was found that the molecule captured Ca ions via the tannic acid's abundant polyphenol groups and induced enamel remineralization in-situ and in-vivo [124].

All these approaches show that the N-terminal statherin sequence is an almost perfect ‘partner’ for the development of remineralization systems. The sequence can be used both for the inhibition of demineralization and for the accumulation of Ca and P ions (frequently F), and to deliver these ions to the locations to be remineralized.

**Fig. 2:**
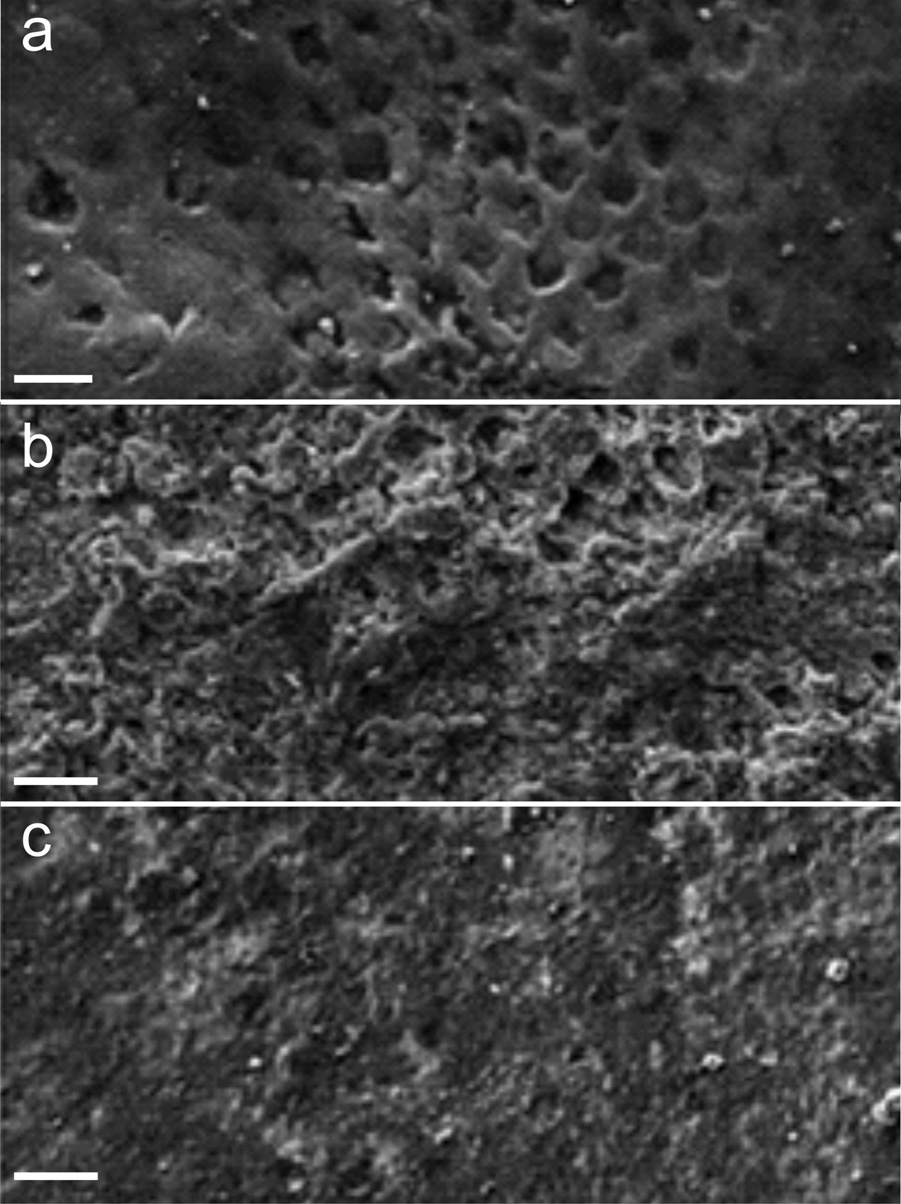
Scanning electron micrographs (SEM) of dental enamel surfaces from extracted premolars. a) Artificially created lesions; the demineralized enamel clearly shows the loss of prismatic (rod) substance (acid etching: 37% phosphoric acid for 20 s, water rinse, drying of surfaces). b,c) Dried samples were treated by applying P_11_–4 to the tooth surface and allowing the substance to diffuse for 5 min. Subsequently the samples were stored in artificial saliva at room temperature (saliva exchange every 14 days) for (b) 1 month with evidence of pore volume reduction and material deposition, and (c) 3 months, showing mineral deposition resembling those on natural enamel surface. Images are taken from [130] and slightly modified (copyrighted and licensed work under Creative Commons CC BY-NC-SA). Scale bars in a,b: 10 µm; in c: 20 µm.

**Scaffold forming P_11_–4 peptides.** The peptide was developed as an agent to promote the enamel remineralization of the early carious lesions without additional calcium and phosphate. Following administration, the β-sheet-forming and self-assembling peptide replaces the degraded enamel matrix with one that promotes in-depth remineralization of the lesion [125]. This is possible due to the sequence of the P_11_–4 peptide (-QQRFEWEFEQQ-), which, under the given local conditions (the presence of cations, high ionic strength, acidic pH), causes the peptide to self-assemble in hierarchical 3-dimensional fibrillary scaffolds (visible as an elastomeric gel) [126,127]. In other words, the β-sheets assemble to ribbons and four ribbons form a fibril. The fibrils in turn create a fibrillary network [127]. Kirkham et al. [127] have shown that these fibrils are arranged in twisted bundles (width: hundreds of nm to a µm; length: several µm), reminiscent of collagen fibers, and that HA mineralized in-vitro along the bundles, indicating sites where HA was nucleated. However, while in-vitro studies of enamel remineralization show that P_11_–4 bundles are adjustable (either parallel aligned or randomly distributed [127]) and that crystallization occurs along these bundles, it has not yet been demonstrated that the bundle alignment is controllable in-vivo [125,128]. The ability to align peptide bundles in one or more directions would certainly facilitate control of the enamel crystallization processes and material properties. It has also been reported that under certain acidic conditions, P_11_–4 cannot completely suppress demineralization (at pH < 4.8) [128] and that the ability to "accumulate" calcium and phosphate ions from saliva is reduced. If, for example, the physiological environment (e.g., pH value, cation concentration) is unfavorable for the formation of the peptide bundles, remineralization is only partially promoted by P_11_–4 [129]. Sindhura et al. [130] have demonstrated the potency of P_11_–4 to remineralize enamel initiating an enamel de- and remineralization cycle (Fig. 2).

P_11_–4 is, to the best of our knowledge, the only iCP remineralization agent that has proven some success in clinical studies. Earlier work reports that P_11_–4 can reverse early occlusal and proximal lesions, [125,126,129,131] while the remineralized lesions showed significantly improved stability for at least 6 – 12 months after treatment [129,131]. In one of the latest reviews, Wierichs et al. [132] analyze clinical studies on the effectiveness of P_11_–4 peptides in the remineralization of early caries lesion. From a total of 911 studies initially identified, 7 studies were assessed for eligibility. These studies were subjected to a meta-analysis, comparing the results of P_11_–4 plus fluoride application vs. fluoride varnish application, as well as for P_11_–4 vs. no treatment. The analysis reveals the following results: (1) The follow-up period was only 6 months with a range between 6 and 12 months. However, it was shown that, depending on the result, treatment with P_11_–4 can be effective in remineralizing caries lesions. (2) There was no evaluation of site effects (e.g. treating the left or the right in split mouth designs [6 studies] but ignoring cross over effects). (3) Of the 7 trials, the bias of 1 study was assessed as low, for 6 it was assessed as high. 6 studies received funds by the manufactures and in 3 studies an employee of the manufacturer was listed as co-author. The results suggest that P_11_–4 could be an option for treating early caries lesions. However, due to the small number of clinical studies (high risk of bias), the short follow-up period and the limiting evidence, the results are unsatisfactory and should be treated with caution.

A further aspect: since P_11_–4 relies on natural remineralization driven by saliva its effectiveness depends on the individual's saliva quality, particularly on its calcium and phosphate concentration, its total mineral content, its pH value, and flow rate [129]. In the case of calcium and phosphate deficiencies, the effectiveness of P_11_–4 could, for example, be increased by adding a calcium-phosphate additive. Still a drawback is the extended amount of enamel repair time limiting the application of P_11_–4 in the clinical setting [79].

Ultimately, more in-situ, in-vivo and clinical long-term trials are needed to confirm and evaluate the findings. It would be of great benefit to identify additional factors/additives that can potentiate the enamel repair process by P_11_–4.

### 3.2 Extrinsic (exogenous) calcium phosphate approaches (eCP)

In the following, some eCP systems are described, which are supposed to lead to an improved remineralization of the dental enamel. Several of them have been clinically tested and commercialized in recent years. Cochrane et al. [3] had categorized the individual approaches into different calcium phosphate systems (crystalline calcium phosphates, unstabilized amorphous calcium phosphates, and stabilized amorphous calcium phosphates). The individual methods are briefly recalled and updated here. In addition, recently developed methods/systems are added to the assessment and the most relevant systems are discussed in more detail.

**Brushite (dicalcium phosphate dihydrate, crystalline).** This compound is one of the more soluble calcium phosphate phases and has been commercialized via dentifrices [133,134]. However, there is a lack of in-vivo studies and the present results from in-vitro work do not reflect the complex biological processes involved in lesion remineralization. In addition, clinical trials are missing. Especially the disease progression of caries has not been studied using brushite [3].

**Functionalized β-tricalcium phosphate (β-TCP, crystalline).** Another crystalline phase is the so-called functionalized β-TCP. In contrast to the other approaches, this compound was not designed to be a substitute for fluorides. Rather, it is used in synergy with fluoride. The functionalization of β-TCP occurs with organic or inorganic components that provide a barrier (adsorbed molecular layer) to prevent premature interactions between calcium (from TCP) and the fluoride. The functionalization also slows down the mineralization when applied with conventional preparations and methods [135]. Functionalization of β-TCP occurs via ball milling with sodium lauryl sulfate (SDS) [136] or via coupling of carboxylates [137]. But also silica or urea were prepared with β-TCP, depending on the reaction conditions and its environment [135].

β-TCP has been commercialized as a calcium and phosphate delivery system (dentifrices and mouthwashes [135]). However, although functionalization is believed to better bind β-TCP to enamel [138], both SDS and carboxylates, but also polymers, copolymers, polyethylenglycol etc., can coat β-TCP, thus leading to a low extent of bioactive calcium and phosphate [139]. This adverse effect of functionalization on remineralization processes suggests the use of non-functionalized β-TCP in toothpastes (without fluoride) [140], even though this contradicts the results of a very recent clinical trial. In this study, Chen et al. have shown that a semiannual varnish application of commercially available 5% NaF varnish with β-TCP (functionalized) is more effective (rate of arresting dentine caries (rADC): 57%) than semiannual use of commercially available 5% NaF varnish without β-TCP (rADC: 42%) [141]. These findings demonstrate that there is still a lack of comprehensive in-vivo and clinical studies to gain more insight into β-TCP-affected mineralization processes.

**Nano-hydroxyapatite (nHA, crystalline).** Nano-hydroxyapatite is considered to be one of the most biocompatible and bioactive materials and has been studied as a material for the reconstruction of dental enamel for quite some time [31,48,142]. For individuals suffering from tooth mineral loss, it has been discussed as an effective anti-caries agent [48,142]. nHA has not only a similar morphology and structure as the apatite crystal within enamel, it has also shown that its small (20 - 100 nm) dimensions are suitable for remineralization of initial submicron enamel erosion [31,48,143,144]. In addition, a comparative study (via pH-cycling, pH = 4 - 7; for 12 days; on demineralized enamel specimens) has shown that nHA promotes remineralization even under neutral pH conditions, whereas micro-HA does not [142].

Nanoparticles have a larger surface/volume ratio than larger particles. They show properties that are not inherent in the larger particles, such as increased adsorption and aggregation potency, or sometimes an amorphous instead of a crystalline state [145], [146], [147]. Therefore, it is not uncommon for nHA particles to have a stronger tendency than larger particles to adsorb to enamel and other locations in the oral cavity. For example, it has been reported that nHA binds to plaque fragments and bacteria, and also acts as a filler to repair small holes and depressions on the enamel surface [145]. The reaction mechanism of nHA with enamel during remineralization is still unclear. Some studies suggest that nHA promotes remineralization through the formation of a new layer of synthetic enamel on the tooth surface and/or in depressions, in small holes and defects [145,148]. Others suggest that nHA acts as a calcium phosphate reservoir (continuous calcium and phosphate ion source) and thus maintains the remineralization process [142]. The latter could lead to a fusion of the aggregated nanoparticles and form a closed layer [143], thereby inhibiting demineralization and enhancing remineralization [142]. On the other hand, Huang et al. [142] have reported that subsurface lesions are not properly remineralized by nHA under pH-neutral conditions. This suggests that nHA might result in less stable enamel layers if pH values are too high.

Several in-vitro studies have demonstrated the potential of nHA in remineralizing enamel. For example, experiments were carried out either via pH-cycling (pH = 4 - 7; for 12 days) [149] or via brushing (for 14 days, twice a day) [150] on surfaces of previously demineralized (at pH 4.5 - 5.0) enamel specimens. The samples were divided into groups that were exposed to either a solution for pH-cycling (fluoride, nHA, negative control) [154 147 149] or a dentifrice (nHA, fluoride dentifrice) for brushing [150]. It was found that nHA remineralizes initial lesions with efficacies comparable [149] or even superior to that of fluoride [150]. A study by Juntavee et al. [151] reports the use of nHA as a gel to remineralize enamel in the cementum-enamel region. Specimens of human molars were coated with nHA gel or fluoride dentifrice (twice daily for 4 min, for 30 days) and the remineralization of the cemento-enamel junction investigated. It was found that nHA was more capable in remineralizing enamel/cementum than fluoride and particularly successful in remineralizing around restoration margins. Although all three studies are based on different approaches, they all find that nHA has either comparable or even better remineralization efficiencies than fluoride. This might indicate that the methodological effects play a smaller role in these studies than the remineralization products and agents used.

In contrast, Esteves-Oliveira et al. [152] have recently demonstrated that nHA is not a 'miracle cure' to stop demineralization in-vitro. Carrying out a study combining pH-cycling (pH = 5 and 7; for 14 days) with brushing periods (twice daily; always after a demineralization phase) enamel specimens (divided into several groups) were treated with fluoride or nHA dentifrice. The analysis showed that groups treated with fluoride reduced mineral loss, while nHA could not significantly decrease the loss of mineral. It is possible that nHA aggregated into larger particles under the given conditions. Such large particles are not able to penetrate into defects or small holes of submicron enamel erosion, which is necessary for remineralization [142]. There is also the question of how large the nHA particles in the dentifrice actually are, how well dispersed they are in the toothpaste's formulation and whether the nHA (zinc-carbonate/hydroxoapatitye; BioRepair [152]) used has any impact on the experiments. Because all factors can influence the solubility of nHA and thus the remineralization behavior of the specimens [151]. Very recently, Juntavee et al. [153] conducted very similar experiments but announced the success of nHA in reducing mineral loss. A difference to the study by Esteves-Oliveira et al. [152] was the use of an alternative nHA toothpaste (Apagard, with pure nHA).

A different (in-vivo) approach was chosen by Najibfard et al. [154]. They prepared so-called "intra-oral appliances" (containing enamel specimens from human permanent teeth), which were adapted to the molars of individuals. The subjects were divided into groups and each group exposed to different products (nHA, fluoride dentifrice), brushing three times a day for 28 days prior to evaluation. Similarly, Ameachi et al. [155] applied the "intraoral appliances" method for their study. In contrast to Najibfard et al. [154] however, with a different assignment for the subjects wearing the intraoral appliances (containing enamel samples [human primary teeth]) and a different nHA dentifrice (Kinder Karex with pure nHA [155] instead of Apagard® with pure nHAP used by Najibfard et al. [154]). Both in-vivo studies report a remineralization quality of the nHAP dentifrice that is comparable to that of the fluoride dentifrice. However, there was no evaluation of site effects; cross-over effects during experimentation seemed to have been ignored in both studies

There are already a number of dental care products on the market (dentifrice: e.g. Apagard, Kinder Karex, BioRepair, ApaCare; rinse products: e.g. Desensine, BioRepair, MicroRepair [31,150,152,[154], [155], [156]]. However, to date, most of the studies on the effectiveness of nHA dental care products have been conducted in-vitro. Some in-vivo studies are also available (see above). But clinical trials and applications are limited, as nHA-based enamel can take several hours to days to form [148]. Very recently, some clinical studies were published that examined the effect of HA on enamel remineralization [157], [158], [159], [160], [161]. However, although all of these trials show HA promoted remineralization, only the studies by Godowitz et al., Badie et al. and Kani et al. were using nanocrystalline HA [159], [160], [161], which, in contrast to (microcrystalline) HA, also demonstrates superiority over fluoride treatments [160]. These trials indicate a clear reduction of the lesion extent [159,160] and an inhibited caries incidence (e.g., of newly erupted teeth) [161]. A somewhat out of line long-term trial was carried out by Grocholewicz et al., comparing the remineralizing potencies of (1) nHA gel with (2) gaseous ozone therapy and (3) a combination of the two [159]. Individuals were randomly divided in three groups and subjected for six months to the three treatment methods with follow-up assessments after one and two years. The study demonstrates a decrease in early caries lesions in all three groups after one year. The caries decline was still clearly visible after the second year. The best results were observed for patients belonging to the combination group (3). Some capability was found for using either nHA or ozone to remineralize enamel surfaces and subsurface lesions. It has been suggested to continue the treatment procedures for a longer period of time in order to achieve caries-free enamel [159]. To the best of our knowledge, the study by Grocholewicz et al. but also the trial by Kani et al. [159,161] are the only clinical studies to date that have tested a nHA supported remineralization over a long period (> 2 years). Ultimately, there is a lack of well-designed long-term clinical trials whose results can confirm the success of nHA before clinicians can use nHA and confidently recommend it as a substitute for fluoride dentifrices and mouthwashes.

**Calcium sodium phosphosilicate (CSP, amorphous) bioglass.** Similar to the crystalline compounds, the bioglass-supported remineralization takes place via the release of ions. The CSP system is described as biocompatible and bioactive. In the oral cavity, CSP releases calcium and phosphate ions that support the natural remineralization of the dental enamel [162]. CSP has been commercialized as NovaMin™ in Oravive toothpaste [31], and has been claimed by manufacturers to successfully remineralize the subsurface lesion [3]. However, both data from in-vitro and in-situ studies are weak and contradicting [163,164]. Moreover, a recent clinical trial using NovaMin™ to repair tooth whitening damage showed no significant effects on enamel remineralization [165]. A very recent review concludes that there is no significant data to support the remineralizing capacity of NovaMin™ when compared to traditional toothpaste [166]. Since CSP could support remineralization in a different form (e.g. more soluble CSP) or in the absence of polymers or other additives (e.g. other toothpaste) further studies both in-vivo and clinical studies are necessary.

**Amorphous calcium phosphate (ACP).** In some publications, ACP is discussed as a possible remineralization compound [3,31]. However, in contrast to other methods, this system seems to be too complicated to be used. A dual-chamber for a calcium and a phosphate salt is required to deliver the salts separately into the oral cavity via fluoride toothpaste [167] (marketed as Enamelon™ [31]). During brushing, calcium mixes with phosphate and forms ACP or (with the fluoride) amorphous calcium fluoride phosphate (ACFP). However, both ACP and ACFP are unstable phases and will transform too fast into stable HA or FA, if the reaction AC(F)P → HA/FA is not controlled (e.g. if the calcium and phosphate salts are not properly functionalized). With little control, calcium and phosphate ions (free or from ACP) are believed to be available just long enough to remineralize enamel surfaces. But both the subsurface lesion plus the surface are not remineralized (HA and FA have very small solubilities) [168]. The control of the reaction time is crucial for a sufficient and durable enamel remineralization using ACPs. More precise, if not under control, the course of the reaction will result in (i) subsurface lesions which are not sufficiently remineralized, (ii) lead to the formation of dental calculus depositions on teeth (because of rapid reactions to HA and FA) and (iii) results in a fluoride underrepresentation for lesion and subsurface lesion remineralization (through rapid FA formation) [3]. For Enamelon™ it was soon suspected that there was insufficient control over the course of the reaction. In addition to in-vitro investigations [169], the system was tested in only one clinical trial, with no clear results of its potency in treatment of caries [170]. Even a review of the latest literature does not provide any new information about an improvement in early caries or early-stage ETW with the use of ACP/ACFP (e.g. Ekambaram et al. [171]). Reviewing Enamelon™, Philip [31] consequently states: "… Considering the limited evidence and better alternatives available, oral products based on the ACP remineralization technology have limited clinical applicability.... "

Very recently Enamelon®, a follow-up version of the older Enamelon™ was introduced. The newly developed formulation does not require a dual chamber and, according to the manufacturer, has been greatly improved in the treatment of early caries lesions [172]. Unfortunately, not many studies have been carried out so far. The new product combines stannous fluoride (SnF_2_) with two polymer complexes of calcium and phosphate salts. Schemehorn et al. report that the formulation promotes a long-term bioavailability of the remineralizing ions [172]. Indeed, the Enamelon® products delivered statistically significant more fluoride to lesions and reduced enamel solubility than TCP, CPP-ACP or other toothpastes containing more or similar fluoride amounts [172,173]. It seems that with Enamelon® a product has been developed with which the reaction rate of calcium and phosphate (and fluoride) to HA or FA can be controlled. However, no manufacturer-independent studies or clinical trials have been carried out to date.

**Fig. 3:**
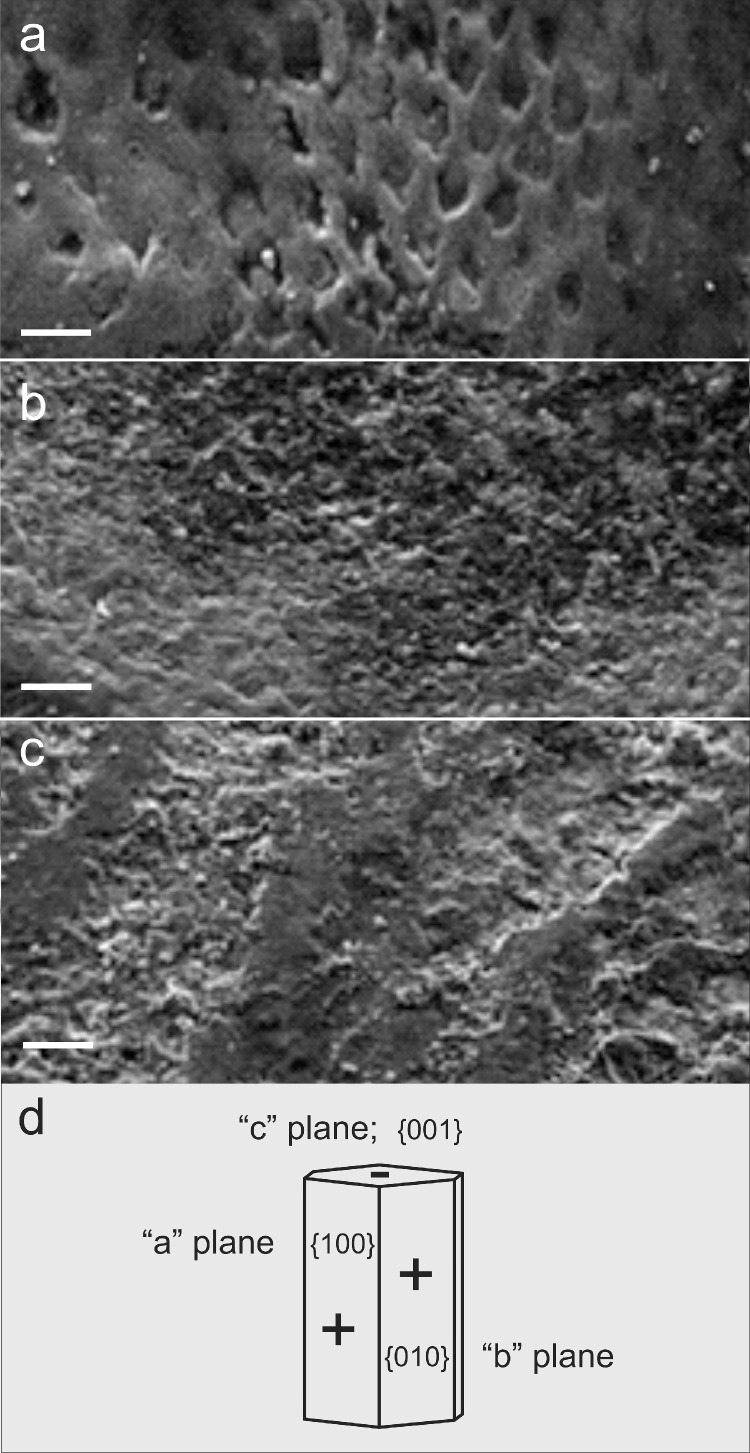
Scanning electron micrographs (SEM) of dental enamel surfaces from extracted premolars (a-c). a) Artificially created lesions; the demineralized enamel clearly shows the loss of prismatic (rod) substance (acid etching: 37% phosphoric acid for 20 s, water rinse, drying of surfaces). b,c) Dried samples were treated by applying CPP-ACP to the tooth surface and allowing the substance to diffuse for 4 min. Subsequently the samples were stored in artificial saliva at room temperature (saliva exchange every 14 days) for (b) 1 month with evidence of strong pore volume reduction and material deposition resembling those on natural enamel surface, and (c) 3 months leading to increased deposits with a distinct wave-like structure. d) Schematic drawing of a HA crystal with developed a-, b- and c planes representing the {100}, {010} and {001} faces, respectively. CPP-ACP absorbs preferentially to positively charged {100} and {010} faces of HA, but less to {001} faces. This hinders the growth of the {100} and {010} faces but promotes the growth of the {001} faces [181,182]. SEM images are taken from Sindhura et al. [130] and slightly modified (copyrighted and licensed work under Creative Commons CC BY-NC-SA). Scale bars in a,b: 10 µm; in c: 20 µm.

**Casein phospho-protein stabilized – amorphous calcium (fluoride) phosphate (CPP-ACP; CPP-ACFP).** This system is a development from ACP. Based on the finding that the solubility of calcium and phosphate ions in living organisms is strongly regulated by proteins, it was proposed to use a protein (or protein fragment) to stabilize ACP or ACFP [174], [175], [176], [177], [178]. The proteins found for this purpose include the caseins in milk and statherin in saliva [179,180]. In milk, for example, casein forms micelles that stabilize calcium and phosphate ions [174,176]. The main reasons for finally choosing caseins are their compatibility and the number of negative charges in individual sequences. The latter is a prerequisite for the quality of both the calcium and phosphate ion stabilization and the stabilization of ACP [175,177,178]. Therefore, CPP, with its larger negative charge and a significantly larger number of phosphorylated amino acids stabilizes (e.g., calcium ions) better than statherin [181]. The advantages of casein and the possibility of sequencing relevant motifs has led to the development of CPP-ACP and CPP-ACFP complexes, which are marketed as Tooth Mousse/MI Paste Crèmes, Recaldent/Trident White sugar-free gum and MI Paste One toothpaste [31,[176], [177], [178]]. Sindhura et al. [130] have recently demonstrated the potency of CPP-ACP in enamel remineralization initiating an enamel de- and remineralization cycle (Fig. 3a-c).

In particular, the sequence –S**p**S**p**S**p**EE– (**p**: phosphor group) has shown that it can stabilize high calcium and phosphate ion concentrations in metastable solution (supersaturated in the presence of solid calcium phosphate phases) [176], at acidic and basic pH [177,182]. The ions were stabilized by the formation of electroneutral ion clusters (hydrodynamic radius: ∼ 1.53 nm and ∼ 2.12 nm for CPP-ACP and CPP-ACFP [in the following just CPP-ACP], respectively) [175,176]. For this compound, Cross et al. found that calcium ions on the surfaces of the calcium phosphate ion clusters interact with the CPP via the negatively charged residues of the peptides [176]. This stabilizing interaction also leads to a controlled growth suppression of these ion clusters. It prevents the clusters from growing to a critical size, which would lead to premature nucleation and phase transformation (amorphous → crystal) [176]. In other words, uncontrolled, rapid growth of the ion clusters can lead to crystallization (mineralization), which predominantly takes place on the enamel surface, but makes access to subsurface lesions more difficult and prevents their remineralization. Finally, there is an equilibrium in the solution between free and CPP-bound calcium, phosphate and fluoride ions. This balance is dependent on e.g., pH, ion concentration, and the presence of competing binding surfaces for CPP [183]. Investigations on the dissociation constant of these casein - calcium phosphate nano-complexes (binding characteristics of calcium and phosphate ions in ACP to CPP) have shown that it is in the mM range [176,184], which indicates that CPPs bind only weakly to calcium and phosphate ions, and thus enable a dynamic equilibrium between free and CPP-bound ions. This scenario, therefore, offers a reservoir of bioavailable ions.

The diffusion gradient enables the CPP clusters to localize even in supragingival plaques. As CPP has a high affinity to HA it will, on entering the lesion, adsorb to the positive charged {100} and {010} faces of HA crystals [185,186] and will hinder these faces to demineralize. This allows, on the other hand, crystal growth only on {001} faces (Fig. 3d), which is the pattern of HA growth in amelogenesis [186].

Low pH conditions, which occur during a cariogenic or acidic attack, strongly influence the solubility of the adsorbed and free ion clusters and result in a promoted release of calcium (fluoride) phosphate ions. This process in turn promotes the remineralization of the incipient lesion through precipitation of the released ions and interrupts demineralization [187]. The remineralization pattern generated by CPP-ACP has been reported to significantly improve the aesthetics, strength, and acid resistance of the remineralized lesions [188].

A large number of studies are now available that demonstrate the ability of CPP-ACP to remineralize enamel. Laboratory, in-situ, in-vivo and a number of clinical trials have shown that the regular use of products containing CPP-ACP promotes the remineralization of enamel and provides adequate caries prevention. The long-term effect of CPP-ACP on remineralization was found to be equivalent to fluorides [189] (or superior [190]), has shown to reduce the amount of *Streptococcus mutans* [191], and rises the salivary buffer capacity [192]. The latter might be an effect of CPP's enzymatic breakdown which results in increased ammonia concentrations, and thus in an increased plaque pH [193,194]. Only recently, an in-vitro study combined with an in-situ clinical study (randomized, controlled, double-blind cross-over design) demonstrated a synergistic effect on the remineralization of enamel by the combination of CPP-ACP + SnF_2_, which was significantly higher than that of the CPP-ACP + NaF, CPP-ACP, NaF and SnF_2_ promoted remineralization [195].

However, some clinical trials come to contradicting results or conflicting conclusions, in particular if CPP-ACP remineralization is compared to fluoride remineralization [31]. The reasons for these controversial outcomes can partly be due to a poor understanding of the CPP-ACP technology. Many trials that did not report any superior added remineralization effect for CPP-ACP compared to fluoride have not taken into account that remineralization patterns produced by CPP-ACP and fluoride are different. While CPP-ACP enhances remineralization of enamel surface and subsurface lesions, fluoride assists in producing predominantly remineralized surfaces [196]. Philip emphasizes that CPP-ACP-remineralized lesions have better aesthetics and higher strength [31]. But they are also more resistant to a subsequent acid attack. Philip also stresses that some inconsistent conclusions arise from considering studies with insufficient statistical power and/or results from studies with a possible conflict of interest between product manufacturers [31]. Finally, colleagues are asked not to neglect the peer review - despite the flood of publications. Obviously, there is still a great need for more independent long-term studies to show whether CPP-ACP products can provide superior remineralization of early caries lesions compared to fluoride-based offerings [31,197].

**Alkali or earth alkali polyphosphates.** More recent developments are so-called polyphosphates*.* They are added to dentifrices to partially replace fluoride. Among the polyphosphates (like sodium trimetaphosphate (STMP), calcium glycerophosphate or hexametaphosphate [198], [199], [200]), STMP is the most effective anti-caries agent to date, and is marketed as Oral-B Pro-Expert toothpaste [31]. STMP is reported to have a dual effect; it hinders demineralization and promotes remineralization [201,202].

STMP (Na_3_P_3_O_9_) strongly binds to phosphate on enamel (which blocks the release of calcium and phosphate from the crystal) and leads to the formation of a layer on the enamel surface that limits acid ion diffusion [203]. It appears, however, that calcium and phosphate diffusion is not affected [199,204]. In addition, STMP can minimize mineral loss even in the presence of low fluoride concentrations, which has been proven in several in-vitro, in-situ and in-vivo studies [202,[205], [206], [207], [208]].

In contrast to most calcium phosphates listed above, several clinical trials with STMP have already been carried out to test the effectiveness. While previous clinical evaluations of the caries preventive effects of STMP produced conflicting results [209,210], a most recent 18-month double-blinded clinical study showed that "sometimes less is more": a 500-ppm low-fluoride dentifrice with STMP was superior to a 1100-ppm fluoride dentifrice in decreasing early caries lesions [201]. However, there is a need for additional long-term clinical studies to ascertain whether STMP can effectively induce remineralization in the absence of fluoride.

### 3.3 Natural products and accelerated remineralization

**Plant products.** In addition to the iCP or eCP systems, some plant products have shown their ability to promote the remineralization of dental enamel.

In particular, galla chinensis has been found to be effective in inhibiting demineralization, improving remineralization, and increasing the effectiveness of fluoride [211,212]. Hesperidin and gum Arabic are other products that have been found to suppress demineralization and promote remineralization [213,214]. However, to the best of our knowledge, none of these products have yet reached the clinical phase.

A recent review on grape seed extract (GSE) as a potential remineralizing agent reported a remineralizing effect (in-vitro) of GSE on caries. Some of the reviewed studies found that GSE is able in mediating enamel remineralization [215]. GSE contains proanthocyanidins (PA), a class of bioflavonoids, that improves the remineralization process in combination with a remineralization agent such as CPP-ACP [216].

**Electrically accelerated and enhanced remineralization (EAER).** An interesting variation to remineralizing agents is the EAER, a recently developed remineralization method by Wu et al. [217]. In contrast to the iCP systems, EAER does not regenerate lost enamel via matrix proteins or the organic capture of calcium and phosphate ions. Rather, it uses the iontophoresis to accelerate the flow of mineralizing ions into the deepest part of the subsurface lesion. In addition, EAER shortens the overall mineralization time compared to agent assist remineralization. The early in-vitro studies are promising; its full potential will, however, depend on results of in-vivo studies [217,218].

## 4 Are non-fluoride approaches suitable for both early caries and early-stage ETW?

Nearly all studies reviewed above aim to replace the traditional (relative unhealthy) fluoride treatment in the near future [44], [45], [46]; and many of the remineralization systems developed in the laboratories can theoretically remineralize both the enamel attacked by early caries and by early-stage ETW [79]. However, most of the clinical studies have been carried out on behalf of caries research (see, e.g., *sec*. about nHA, CPP-ACP and P_11_–4). Clinical trials for ETW research have, to the best of our knowledge, not yet been commissioned.

On the other hand, it must be emphasized that a clinical study on ETW is difficult to implement. Such a study could only be performed for a specific group of ETW patients (ETW is ranked to the extent of tooth wear [42]) and it would be necessary to include all available treatment tools for this group of patients (e.g., avoid acid and mechanical influences, eat calcium and phosphate-rich foods such as cheese etc. [219,220]). A task that is too laborious. However, a fluoride-free calcium phosphate toothpaste clinically tested for early caries lesions that assists in remineralizing dental enamel is definitely an additional tool to consider when dealing with early-stage ETW [219].

Various studies have demonstrated that some of the above discussed non-fluoride remineralization systems are not only effective in caries prevention, but can also help to stop an early-stage ETW [219,221]. For cases of early-stage ETW, Ionta et al. [222] have shown in a randomized, single-blind in situ/ex vivo study that the use of calcium phosphate toothpaste reduced the loss of dental enamel, but had no effect against exposure to acids and brushes. In an earlier study, Rees et al. [223] have reported that a mousse containing CPP-ACP offered some protection from erosive drinks. But it showed no superiority over fluoride toothpaste. In addition, Munoz et al. [224] have shown that remineralizing toothpastes such as Enamelon™ (an ACP approach, see above for details) increase the surface hardness of teeth that have been exposed to acidic substances and that these toothpastes are more effective than conventional toothpastes containing fluoride alone.

In summary, it can be stated that the developments in the field of non-fluoride remineralization systems represent a major step forward for non-invasive dentistry. These systems have been studies primarily for/with caries patients. With regard to ETW, the calcium phosphate systems (perhaps in combination with fluorides) are ideally suited as one tool among many to counteract the progression of the ETW.

## 5 Conclusions

The distant goal of modern non-invasive treatment of early (non-cavitated) caries lesions and early-stage (submirometer) ETW is to replace fluorides with non-fluoride enamel remineralization systems. Fluoride treatment is still the prominent 'tool' in dentistry. However, a number of systems are currently being developed that improve the effectiveness of fluoride and thus reduce the fluoride concentration required for remineralization or even make the use of fluoride redundant. This drastically minimizes the potential risks associated with frequent fluoride treatments/intakes and overexposure to fluoride (especially in children), which can result in the development of dental fluorosis or to an exacerbation of an existing fluoride syndrome.

Currently, two pathways are being pursued in the development of non-fluoride enamel remineralization approaches: the eCP and the iCP remineralization systems. The eCP systems work due to their external supply of calcium and phosphates (and fluoride if desired) to the enamel crystals to be remineralized. Of the systems discussed in the article, the casein phospho-protein - amorphous calcium (fluoride) phosphate (CPP-ACP; CPP-ACFP) and the nanocrystalline hydroxyapatite (nHA) are currently the most advanced systems. Many laboratory reports and a number of clinical studies confirm that these systems have strong anti-caries properties and are effective in caries prevention. It has also been shown that enamel of early-stage ETW can be successfully remineralized with CPP-ACP, and protected from erosive beverages. In the meantime, these systems have been successfully tested in the absence of fluoride, which make them particularly suitable for risk groups of patients (the future main recipients of non-fluoride products). The systems β-TCP or STMP have been subjected to clinical trials as well, but only in combination with fluorides and with less success compared to fluorides alone. iCP systems have joined the non-invasive remineralization systems. In terms of their nature and function, these systems are very similar to the biological systems of enamel formation and do not require the external addition of calcium phosphate for remineralization. They capture calcium and phosphate from saliva, while fluoride can be added to the systems. In general, development work and clinical studies of these systems are still in their infancy. Currently, only one of the systems discussed in the article is a promising candidate for future dental treatments. This system, the so-called P_11_–4, is a self-assembling peptide that mimics the properties and function of amelogenin. However, there is still a long way to go. In particular, unbiased studies and clinical trials are required before dentists can use this system with confidence. A disadvantage of the iCP systems in general is their extended amount of enamel repair time (sometimes days/weeks), which will limit the application of these materials in the clinical setting.

In summary, the non-fluoride enamel remineralization systems under development show promise. However, the currently available clinical evidence for most of the remineralization systems discussed here is either absent, poor, or inconclusive. Well-designed (randomized) clinical studies are crucial in order to clarify whether these remineralization approaches offer an additional advantage for dental applications over conventional fluoride remineralization. These studies are required in particular for the products that are already on the market.

## CRediT authorship contribution statement

**Bernd Grohe:** Conceptualization, Project administration, Writing – original draft, Writing – review & editing. **Silvia Mittler:** Conceptualization, Funding acquisition, Writing – review & editing.

## Declaration of Competing Interests

The authors declare that they have no known competing financial interests or personal relationships that could have appeared to influence the work reported in this paper.

## Appendix Supplementary materials

Image, application 1
