# Supplementary material for: Advanced non-fluoride approaches to dental enamel remineralization: The next level in enamel repair management
Source: Biomater Biosyst. 2021 Oct 29;4:100029. doi: 10.1016/j.bbiosy.2021.100029 (PMC9934497; doi:10.1016/j.bbiosy.2021.100029)
Supplement: Supplementary file 1 [file mmc1.docx]

Supplemental Material

Advanced non-fluoride approaches to dental enamel remineralization: the next level in enamel repair management

Bernd Grohe^a*^, Silvia Mittler^b,c^

^a^ Lawson Health Research Institute, St. Joseph’s Hospital, London, Ontario, Canada, N6A 4V2

^b^ Department of Physics & Astronomy, University of Western Ontario, London, Ontario, Canada N6A 3K7

^c^ Department of Chemical and Biochemical Engineering, University of Western Ontario, London, Ontario, Canada N6A 5B9

**Short tutorial/refresher on the dental enamel formation, its breakdown and remineralization**

**1. Dental enamel and its structure**

Dental enamel is the hardest tissue in humans (harder than, e.g., dentin, cementum and bone) [1]. Fully formed enamel consist of approximately 95 - 96 wt.% non-stoichiometric carbonated hydroxyapatite (HA; Ca_5_(PO_4_,CO_3_)_3_(OH), without taking Na^+^, Mg^2+^, Cl^-^, and F^-^ traces into account), ~ 1 wt.% organic material, and ~ 3 - 4 wt.% water [2,3]. Enamel, which is covering the entire crown of the [tooth](https://www.britannica.com/science/tooth-anatomy) (Fig. S1a), is a composite material consisting of densely packed and intertwined (or parallel arranged) mineral rods bonded by proteins (Fig S1b) [4]. These proteins are part of a thin (~ 0.1 µm) interfacial layer system that separates the rods and consist mostly of water. The rods (diameter ~ 5-6 µm), in turn, are made up of protein-bound (particularly enamelins) HA fibers that run parallel to or at different angles to the rod direction (Fig. S1c,d). These fibers have a width of ~ 30 nm and a length of ~ 70 nm [4,5]. Enamel thus inherits (a) a high level of hardness and rigidity from its structured main component, the relatively hard HA (in the direction of the mechanical stress when chewing); while (b) the soft and ductile organic/aqueous phase between the crystals and the rods reduces the risk of catastrophic failure. Thus, human enamel can withstand high loads with good fracture resistance and structural reliability. In addition, due to the structuring of the material, enamel can withstand chemical erosion for some time (e.g. food acids: softening of the top layer: ~ 10 µm in ~ 30 min) [6].





**Fig. S1.** Schematic representation of the microstructure of dental enamel. a) Structure of the tooth ([7]: Image modified. Copyrighted and licensed work under Creative Commons CC BY 4.0). b) Cross section through the dental enamel. Please note the densely packed keyhole-shaped (top of the tooth) mineral rods, which run parallel (or intertwined) to the direction of tooth growth and which are bound by proteins. c) Scanning electron microscopy (SEM) image of a brushed tooth surface ([8]: Image modified. Copyrighted and licensed work under Creative Commons CC BY-NC 4.0). d) A section of enamel rods (rotated by ~ 90° to the picture in b) shows that individual rods are built up of fibers (HA: protein-bound hydroxyapatite) that run parallel or at different angles to the rod direction ([9]: Image modified. Copyrighted and licensed work under Creative Commons CC BY 3.0).

**2. Enamel biofilm**

**2.1. The acquired pellicle and the bacterial layer; components and structure**

Enamel is always in contact with saliva. Immediately adjacent to the enamel, a biofilm is formed. This film is composed of the so-called acquired pellicle and the bacteria-containing plaque [10]. The acquired pellicle, an interfacial layer between enamel and plaque is bacteria-free and covers the oral hard and soft tissue. It consists of lipids, mucins, glycoproteins, proteins (including several enzymes) and their respective derivatives [11,12]. The pellicle forms by adsorption of salivary proteins that are affine to enamel and by intermolecular protein-protein interactions establishing globular structures (Fig. S2). The thickness of the pellicle varies largely between ~ 1 nm (shortly after, e.g., tooth brushing) and 900 - 1000 nm after 24 h undisturbed pellicle formation [13,11]. In direct contact with the acquired pellicle, a transitional layer of bacteria forms, which shows a decreasing bacterial density with increasing distance from the tooth surface (Fig. S2). During the plaque formation process, different types of bacteria adhere, aggregate, proliferate and generate a mature film (dental plaque). At a sufficient distance from the tooth surface, bacteria disperse and spread to colonize new areas [10]. Bacteria in direct contact with the pellicle are able to penetrate the protein layer [10].





**Fig. S2.** Scheme of the oral biofilm. The pellicle (bacteria free) forms by adsorption of salivary proteins on enamel. Protein-protein interactions result in a globular structure of the pellicle and to a dense "basal layer" of proteins, which are in direct contact with the dental enamel. Adjacent to the pellicle, a transitional layer - the plaque - forms by adherence and aggregation of bacteria. This layer exhibits a decreasing bacterial density with increasing distance from the tooth surface, in which bacteria in direct contact with the pellicle are able to partially penetrate the pellicle.

**2.2. Biofilm formation process; adsorption and bacterial build-up**

The biofilm formation is a two-step process in which the pellicle proteins first interact and adsorb to the dental enamel, followed by the adsorption of bacteria onto the pellicle-covered enamel. The mechanisms of pellicle formation are based on Gibbs' law of free energy because, regardless of the mechanisms and kinetics during the pellicle formation process, adsorption can only take place if the Gibbs' free energy *G* decreases [10,14]:

*ΔG_ads_ = ΔH_ads_ – TΔS_ads_ < 0*  (1)

where *H*, *S* and *T* are the enthalpy, entropy and temperature, respectively, and *ΔG* indicates the overall change of the thermodynamic functions resulting from the adsorption process. Protein adsorption (and the associated reduction in free energy) is therefore a net result of various interactions between the system components (proteins, dental enamel, water molecules, ions, etc.) and reflects processes such as the desolvation and dehydration (in part) of the solid/liquid interface, the redistribution of charged groups in the interfacial layer, the structural rearrangements in the protein molecules, protein-protein interactions and the reorganization of the globular pellicle. In brief, changes in entropy and enthalpy mirror the reorganization processes by which water accommodates the solutes und proteins [14-16]. The origin of these processes are protein enamel interaction forces (on a length scale of up to 100 nm [10]) such as Lifshitz - van der Waals forces, Lewis acid-base forces, electrostatic forces and entropically based effects, all contributing to a decrease in *ΔG* [14,17]; with a predominant contribution from entropic mechanisms [17].

As the pellicle continues to form and maintains, the adsorption of bacteria and plaque build-up begins. With some types of bacteria, adsorption occurs within seconds (reversible), but typically it takes minutes (irreversible) [17]. The adsorption process occurs via Lifshitz - van der Waals and Lewis acid-base forces, entropic contributions and as a result of electrostatic-double layer interactions (Fig. S3 top). The entire process reduces the free energy (*ΔG*; equation 1) as a function of the distance between the bacteria and the enamel surface (~ 10 – 100 nm; Fig. S3 bottom; floating position). In order to make the adhesion permanent, some bacteria send out tethers (fibrils/fimbriae; see Fig. S3: orange zig-zag lines) formed e.g. via extracellular-polymeric-substances. They "pierce trough" the potential energy barrier and thus reach the deep primary energy minimum, a few nm adjacent from the enamel surface (Fig. S3 bottom; tether-coupled). The stabilization of the bacterial-surface-adhesion depends on van der Waals forces, calcium and protein bridging, specific recognition sites, hydrophobic interactions and entropic contributions [10,15-17]. The process is associated with a progressive bond-strengthening due to the removal of interfacial water, structural changes in proteins, and re-arrangement of bacteria to expose favorable adhesion sites, like, e.g., fibrils/fimbriae towards the enamel surface [17].


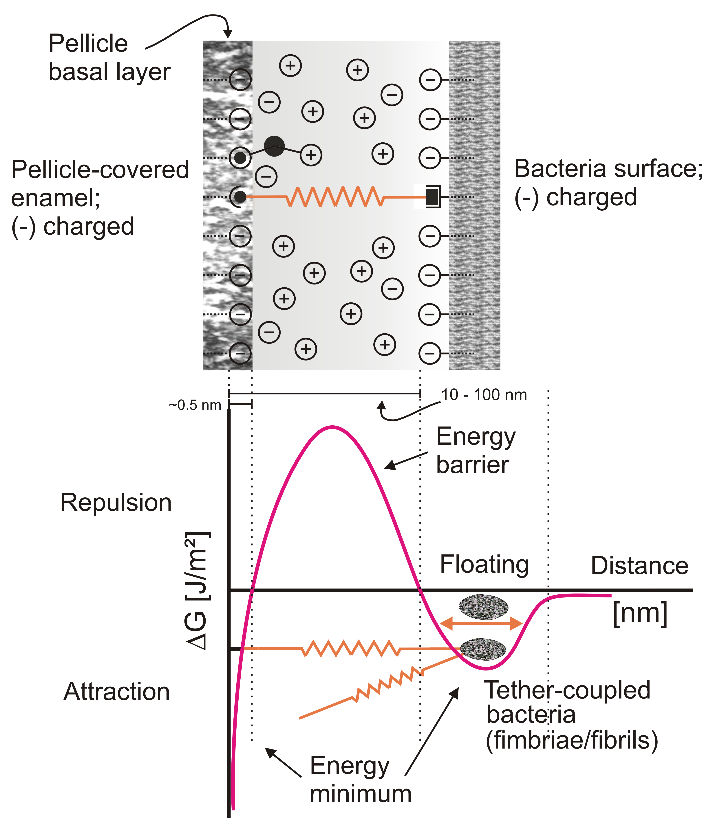


**Fig. S3.** Scheme of bacteria-surface interactions (zig-zag lines: bacterial tethers or fibrils/fimbriae) Top: Scheme of the structure formed by the pellicle-covered enamel and the bacterial surface. Bottom: The interfacial Gibbs free energy of adhesion *ΔG* as a function of the separation distance between a negatively charged bacterium surface and the negatively charged pellicle-covered enamel surface in an aqueous solvent (saliva) of moderate ionic strength (note: both bacterial cell surfaces and enamel surfaces can become positively charged depending on the pH and ionic strength). While Lifshitz - van der Waals interactions are virtually always attractive, electrostatic double-layer interactions are usually repulsive resulting (according to the Derjaguin, Landau, Verwey, Overbeek (DLVO) theory [17]) in an energy minimum (10 – 100 nm from the enamel surface). Here the bacteria are temporary held and subsequently stabilized (tether-coupled) by bacterial fibrils/fimbriae that interact with the enamel surface or pellicle proteins adsorbed to the enamel surface.

**3. De- and remineralization of enamel**

In a healthy oral cavity (with adequate oral hygiene), enamel mineralization is relatively stable, with a dynamic equilibrium between demineralization and remineralization processes at the interfaces between tooth-acquired pellicle and plaque-saliva. In this situation, the amount lost by demineralization D is approximately the amount of mineral deposited by remineralization R: D ≈ R. In cases in which demineralization is pronounced (D > R), such as in caries or high consumption of acidic beverages, processes will lead to lesion formation (“white spots”) or enamel surface softening. However, if D < R, defects present in enamel will be remineralized by calcium and phosphate of saliva or by remineralizing agents [3,18]. The following paragraphs briefly describe the effects of caries and erosive tooth wear processes on the enamel and its demineralization. More details about the remineralization of enamel are discussed in separate chapters (3.2. and 3.3.).

**3.1. Demineralization and degradation of enamel**

**3.1.1. Impact of caries**

Caries is a disease caused by bacteria fermenting foods (carbohydrates) in the dental plaque. The fermentation process produces organic acids (e.g. lactic, acetic, formic and propionic) that can attack and dissolve tooth mineral. So-called cariogenic bacteria (e.g. mutans streptococci and the lactobacilli species) are essential to the disease process. Some of these bacteria are aciduric; they can live in their metabolically produced acidic environment [19]. When the organic acids are produced by the bacteria they readily diffuse in all directions, penetrate the acquired pellicle (see Fig. S2) and diffuse into enamel (or dentine and the underlying tissue). In the enamel, the acids encounter acid-soluble mineral (HA) and begin slowly to dissolve it (pH ~4 - 5) [20,21]. At this stage, caries lesions will form. If this process progresses over months or years cavities will form as a result [1,22].

**3.1.2 Erosive tooth wear (ETW)**

Erosive tooth wear (ETW; also known as dental erosion; DE) of enamel is caused by chemical degradation and mechanical wear, without the involvement of bacteria. ETW is a combination of two or more of the following mechanisms, almost always starting with an acid attack [6].

**Acid attack (erosion)**. The term erosion is sometimes used in dentistry to describe the superficial loss of dental hard tissue due to an acid-based chemical process without the involvement of bacteria. The acidic chemicals involved in degrading enamel can be intrinsic or extrinsic. The former are mainly gastric substances caused by recurrent vomiting or regurgitation processes [23]. The extrinsic acids result mainly from the consumption of acidic foods and beverages, with acidic beverages (pH 2.6 - 3.6) being the main cause of erosion. Enamel losses in the range of 3-100% were reported, with a higher prevalence being found in younger people [6,24,25]. The physicochemical mechanisms of chemical erosion by ETW are similar to the demineralization processes in caries. In contrast to caries, however, the effect of acid on the enamel is short-term and at low pH values (as low as pH ~ 2 - 2.5) [6,26,27]. Paragraph 3.1.3. (below) takes a closer look at these processes.

**Attrition. The term describes the wear and tear of teeth caused by tooth-to-tooth contact.** It occurs between two teeth as well as a tooth and enamel fragments generated due to wear/chipping. Signs of attrition are usually found on the occlusal surfaces or incisal edges of teeth [28]. In particular, grinding of teeth (bruxism) wears out the tooth enamel drastically through abrasion [6].

**Abrasion.** Abrasion describes processes of wear caused by the mechanical friction of exogenous material that is forced over the surface of the enamel, for example by cleaning teeth and chewing [29]. Common exogenous materials include toothpaste/brush and food particles. For example, over-brushing of teeth for the purpose of teeth whitening can lead to severe wear of enamel [6].

**Abfraction.** The term defines wear processes that are caused by mechanical stresses due to flexure of a tooth under heavy lateral loads. This can lead to displacement or fracture of enamel prisms at the cemento-enamel junction [30] (at the root-crown junction, see Fig. S1a). High stresses are concentrated in the cervical region (margin above the root-crown junction), where the enamel layer is thin. In addition, non-carious cervical lesions can form [31].

Usually, ETW starts with an acid attack and softens the enamel. Enamel is therefore more susceptible to mechanical forces such as attrition or abrasion (see Fig. S5). The acid attack can start a cycle in which erosion and abrasion/attrition act simultaneously or successively. If this cycle is not broken, the tooth will decay or even be lost [6,32,33]. In dentistry, the so-called ‘Basic Erosive Wear Examination (BEWE)’ categorizes the extend of tooth wear, helping dentists in risk management [34].

**3.1.3. Erosion processes in caries and ETW: a closer look**

During the development of caries (a relatively long-lasting process), cariogenic organisms (e.g. mutans streptococci) present in plaque initially synthesize intracellular polysaccharides locally. As a by-product, metabolic acids are produced that lower the pH in the vicinity of aggregated and packed bacteria below pH 6. The acid-producing organisms can keep this pH for several hours in the presence of buffering saliva (pH ~ 7) [19,20]. Meanwhile, the number of sites of acid-producing bacteria in the plaque increase, which is accompanied by an increase in fermented carbohydrates and a decrease in pH to ~ 5.5 due to the higher acidity [20]. In the processes that follow, the rate of polysaccharide synthesis and thus the acid production on the tooth surface increase sharply. This favors the dissolution of calcium and phosphate from the enamel locally, demineralization takes place, and dental enamel (mineral) is partially lost. The pH on the tooth surfaces drops further to values of pH ~ 4, which can happen within a few minutes. These processes are favored when sugar and/or acidic food is present [20]. As mentioned above, at this stage caries lesion can form, and if not treated the disease will result in cavities within months or years [1,22].

In ETW-induced chemical degradation, basically the same processes are observed, with the exception that bacteria are absent and the process is relatively short-term. In addition, pH values can be much lower (around pH 2 - 3) than those measured in carious demineralization processes (pH 4 - 5). In caries, for example, the ingestion of sucrose by a person with normal salivary flow results in a decrease in pH from 7 to 4.5 within 5 minutes and a subsequent increase to pH 7 within the next 25 minutes [35]. In ETW, however, the consumption of acidic foods and beverages such as vinegar, apples, citrus fruits and sodas (containing, e.g., damaging citric acid and phosphoric acid) lowers the pH. These food acids increase the acidity even further than the processes in caries, resulting in pH values as low as pH ~2.5 in a relative short time. [24,25]. It seems obvious that remineralization of enamel that has been degraded by such an acid attack can be very tedious and sometimes not feasible.

Microscopically the following phenomena can be observed. As the acid concentration rises, the acid molecules increasingly diffuse in all directions; also through the acquired pellicle where they encounter the tooth surface [20,21]. The acid molecules diffuse into the porous subsurface enamel (sites of pH ~ 7) and then continue on their way to dissociate and enrich the water between the HA crystals, especially the water within the interfacial layer system surrounding the prismatic rods (see Fig. S1). When the acid molecules reach the HA crystals they dissolve them and set Ca and P ions free into the solution surrounding the crystals (Ca, P can be chelated with acid molecules). Some Ca and P ions can also diffuse out of the tooth (Fig. S4a) [3,19,35]. For the latter, high concentration gradients of Ca and P between the subsurface and the surface layers are responsible. They are the driving force for the diffusive Ca and P transport [36]. The dissolution processes preferably start at susceptible sites on the crystal surface, mostly at the Ca-rich edges and the hexagonal ends ({0001} crystal faces), and lead to internally hollowed out crystallites with rounded corners (Fig. S4b) [37-39]. The hollowing out of the crystals results from the relatively high leachability of the hydroxyl (OH) ions, which form the c-axis perpendicular to the {0001} face of HA (Fig. S4c) [3,39,40].





**Fig. S4.** Demineralization of HA in enamel. a) SEM micrograph of acid-etched dental enamel; etching of crystals of the interprismatic (IP) layer system is preferred over prism crystals ([41]: Copyrighted and licensed work under Creative Commons CC BY-NC-ND 4.0. b) Schematic drawing: acid-etching results in rounded crystal edges (calcium loss) and the dissolution of the core by ‘leaching‘ of hydroxyl ions along the crystallographic c-axis (for remarkable details see [39], see also chapter 3.3.). c) Scheme of a {0001} HA crystal face: hexagonal arrangement of calcium and phosphate ions perpendicular to the c-axis (the 'hydroxide screw axis').

If this process is not interrupted, the development of the macroscopically visible white patches (lesions) on tooth surfaces is an indication of a relatively long exposure to acid and areas of calcium deficiency. These are the regions that are most susceptible to acid attack due to the substitution of phosphate ions in the HA crystal by carbonate ions, producing defects and calcium deficient regions [19].

Eroding enamel can form a three-layer structure; a) a layer of irreversible bulk loss, b) a softened layer and c) a transition layer between the softened/outer layer and sound enamel (see Fig S5) [6,41]. After the loss of the first layer, the enamel begins to soften, which is limited to a “depth” of 1 – 15 µm [3,42]. However, the softened layer is only a partially demineralized structure (remineralization is possible under suitable conditions), with the remaining tissue acting as a scaffold [23]. Only continuous acid attack can totally dissolve the softened layer and result in its total loss. At this stage, acid penetrates along the inter-rod sheaths, which induces the dissolution of subsurface (sound) enamel and forms a transition layer (Fig. S5a). Crystals along the inter-rod sheaths will be demineralized while crystals within the prisms are hardly affected [42]. If the process is not interrupted at this point, rods (prisms) will partially or completely disappear [3]. The lesion (visible as white spots) resulting from subsurface demineralization has a low internal mineral content, while the developed surface layer covering the lesion has a high mineral content [43]. The surface layer is porous and has a thickness of approximately 20 µm. The depth of the lesion may vary between 20 – 1000 µm [3,42].

When – as usual in ETW – an acid attack is combined with mechanical wear such as attrition or abrasion, the rate of enamel loss increases further. In these processes, the chemical degradation softens the enamel surface, while the mechanical impact scrapes the softened enamel layer (Fig. S5b,c). If this cycle of softening/scraping of the enamel is not broken, the tooth will decay or even be lost [6,32,33].


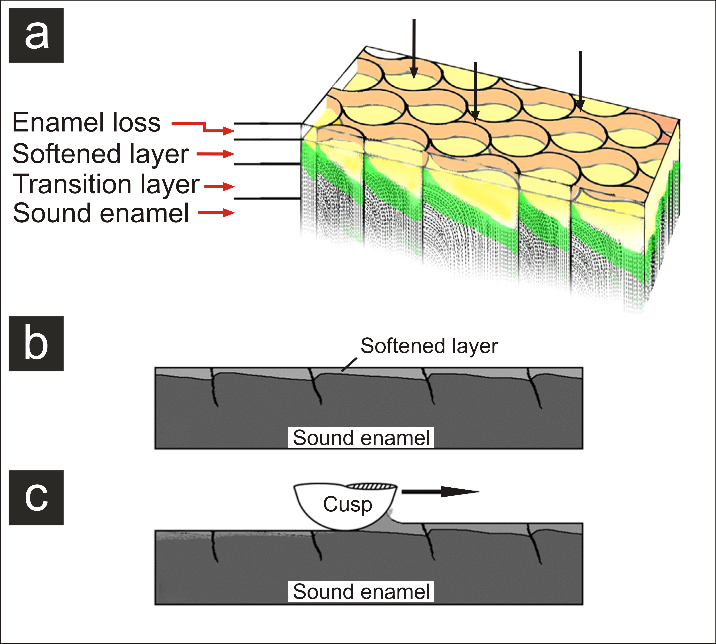


**Fig. S5.** ETW impact and loss of enamel: erosion and mechanical wear. a) Scheme of the formation of an enamel multilayer structure due to erosion. Black arrows: direction of acid attack. b,c) Scheme of the mechanical impact of scraping on a softened enamel layer. (both schemes: [6] - Copyrighted and licensed work under Creative Commons CC BY-NC-ND 4.0, (a) slightly modified).

**3.2. Remineralization of enamel**

As described above, the chemical (acid) induced demineralization/degradation of early caries and early-stage ETW begins at the atomic level (at HA crystal surfaces) within dental enamel and, if not stopped, continues until cavities are formed. There are many possibilities to intervene in this continuing process to interrupt or reverse the progress. For nature, remineralization is the repair process of early (non-cavitated) caries lesions on the surface of the tooth (incl. subsurface lesions) or enamel of early-stage ETW. However, this is not a one-time process. Since the acid attack is episodic and the destruction occurs more or less continuously, repair measures are constantly required; i.e. the environment needs to be continuously monitored and adapted in order to maintain the natural repair process (remineralization). In addition, large fluctuations in mineral and organic composition of enamel are likely to result in local rate-variations of demineralization and remineralization processes [19,40].

Remineralization relies on calcium and phosphate ions to build-up a new surface, with new enamel material growing on existing crystal remnants in subsurface lesions that remain intact after demineralization [19]. Remineralization will start when the transport of Ca and P from the subsurface to the surface layers (due to the high concentration gradient) is almost complete and the pH has risen to ~ 7. The resulting supersaturation of Ca and P ions in these surface layers will then lead to the precipitation of calcium phosphates [36].

Under optimum (healthy) conditions, these processes are induced when the salivary flow increasingly accumulates calcium and phosphate ions in demineralized areas at a then prevailing pH between 6.75 and 7.25 (if fluoride is present even at lower pH values, see main manuscript sec. 2). At peak, the Ca and P concentration in demineralized lesions can be 30 times higher than in supersaturated (already remineralized) areas [20,36]. For these accumulation processes to occur, an ionic and protein-rich composition of saliva is important, as it promotes its ability to buffer reaction processes (e.g. by bicarbonate, phosphate, carbonic anhydrate, basic alkaline proteins) and to control the solidification during remineralization (e.g. by proline-rich glycoproteins, statherin [and peptides thereof], mucins, calcium, phosphate) [44,45]. The adjustments of the solution parameters through changes in pH, ionic strength, the proteins present etc. finally tailor the accumulation reactions and avoid super or undersaturation of the surface layers with Ca and P. For example, high Ca and P supersaturations at the surface layers could lead to a high precipitation rate of calcium phosphates and thus close pores that have to remain open for the diffusion of Ca and P from the demineralized subsurface to the surface layers [21]. High precipitation rates also result in calcium phosphates that do not correspond to the stoichiometry for HA (Ca/P = 1.67). Some authors report that supersaturation should be close to saturation of HA precipitation in order for the precipitation rate to be slow and controllable for proper remineralization [46,47]. Early work by Nancollas and coworkers suggests that remineralization, which is essentially a surface-controlled reaction, produces numerous "seeds" of hydroxyapatite-like material. These initial nuclei are probably an amorphous precursor phase that quickly transforms into crystalline apatite (amorphous calcium phosphate 🡪 octa calcium phosphate 🡪 HA) [48-50]. A thorough stabilization of the precursor phase is assumed to be critical for remineralization of the enamel surface - the slow conversion gives the calcium phosphate precursor and the resulting crystallite phases time to generate a compact surface. If the phase change to HA is too fast, fragile and porous surface layers are created that cannot withstand acid attacks for long. For example, magnesium ions appear to stabilize the calcium phosphate precursor. In addition, a synergistic effect between magnesium and adenosine triphosphate (conversion-delay of ACP to HA) has been reported in this context [21,51,52]. It should be pointed out again that the suppression of demineralization cannot be equated with the promotion of remineralization or that the suppression of demineralization "... is an essential function for promoting dental remineralization ... " [53]. De-and remineralization are two fundamental parts of enamel mineralization; both processes are reacting in two opposite directions that influence each other, as described in detail above.

**3.3. De- and Remineralization at the nano-scale.**

Using high resolution and highly sensitive methods, very recently some laboratories have made advances in the analysis and understanding of the de- and remineralization processes of enamel (HA) crystals [39,54,55]. The defect that can be observed in the central area of the enamel (HA) crystals (the central dark line (CDL [56]), along the 'hydroxide screw axis') is crucial in these processes, since enamel crystals dissolve starting from the center during acid attacks (e.g. due to caries; see also sec. 3.1.3. and Fig. S4b,c) [37-39,57]. Reyes-Gasga & Brès [54] have reported that, starting from the hexagonal HA unit cell, a systematic destruction of the enamel crystals occurs, with a central lesion initially along the [$11\overline{2}1$]-direction on the basal (0001) planes which then develops anisotropically along the [0001]-direction transversely to the crystal [38].

A complete explanation for the anisotropic crystal dissolution has not yet been presented. Neither the reason for the anisotropic dissolution nor its impact on the mineralization/demineralization process are currently fully understood. However, recent studies by Gordon et al. [58] and Yun et al. [55] have found high concentrations of Na^+^ and Mg^2+^ ions in the center of enamel crystals. DeRocher et al. [39] specified these findings and demonstrated the existence of two 'nanometer layers'. These layers were enriched with Mg^2+^ ions, which flanked a core that in turn was enriched with Na^+^, F^–^, and CO_3_^2-^ ions. DeRocher et al. describe this core as a sandwich core, which is surrounded by a shell with a low concentration of substitution defects [39]. Based on measurements and a mechanical model, it was predicted that the chemical gradients favor an increase in the remaining stresses [39], which could ultimately favor the anisotropic carious dissolution [54]. What does this mean for the remineralization process? Yun et al. [55] suggest that this central region, the crystal defect-enriched CDL within hydroxyapatite nano-crystallites, provides a pathway for ion exchange during demineralization and remineralization. It has also been shown that organic-rich precipitates and large-angle grain boundaries are more susceptible to acid attack than small-angle grain boundaries [55]. Yun et al. [55] attribute this to the lower crystallinity in these regions. These regions are suggested to be in turn one of the first to be remineralized.

**References**

[1] A.S. Cole, J.E. Eastoe, Biochemistry and oral biology*,* Butterworth, London, UK, 1988.

[2] J.C. Elliott, D.W. Holcomb, R.A. Young, Infrared determination of the degree of substitution of hydroxyl by carbonate ions in human dental enamel, Calcif. Tissue Int. 37 (1985) 372-375. https://doi.org/10.1007/BF02553704.

## [3] [J. Arends](https://www.sciencedirect.com/science/article/pii/0022024881900609#!), [J.M. Ten Cate](https://www.sciencedirect.com/science/article/pii/0022024881900609#!), Tooth enamel remineralization, J. Crys. Growth 53 (1) (1981) 135-147. https://doi.org/10.1016/0022-0248(81)90060-9.

[4] J.W. Pro, F. Barthelat, Discrete element models of tooth enamel, a complex three-dimensional biological composite, Acta Biomaterialia 94 (2019) 536–552. https://doi.org/10.1016/j.actbio.2019.04.058.

[5] B. Kerebel, G. Daculsi, L.M. Kerebel, Ultrastructural studies of enamel crystallites, J. Dent. Res. 58 (spec. issue B) (1979) 844-851. https://doi.org/10.1177/00220345790580023701.

[6] [Y.-Q. Wu](https://www.sciencedirect.com/science/article/pii/S2405451817300405?via%3Dihub#!), [J.A. Arsecularatne](https://www.sciencedirect.com/science/article/pii/S2405451817300405?via%3Dihub#!), [M. Hoffman](https://www.sciencedirect.com/science/article/pii/S2405451817300405?via%3Dihub#!), Attrition-corrosion of human dental enamel: A review**,** Biosurface and Biotribology 3 (4) (2017) 196-210. https://doi.org/10.1016/j.bsbt.2017.12.001.

[7] B. Blaus, Blausen.com staff, Medical gallery of Blausen Medical, 2014. WikiJournal of Medicine 1 (2). https://doi.org/10.15347/WJM/2014.010.

[8] A. de Almeida Neves, R. de Almeida Castro, E. Tavares Coutinho, L. Guimarães Primo, Microstructural analysis of demineralized primary enamel after in-vitro tooth brushing, Pesqui. Odontol. Bras. 16 (2) (2002) 137-143. https://doi.org/10.1590/S1517-74912002000200008.

[9] T. Sui, M.A. Sandholzer, N. Baimpas, I.P. Dolbnya, G. Landini, A.M. Korsunsky; Hierarchical modelling of elastic behaviour of human enamel based on synchrotron diffraction characterization, J. Struct. Biol. 184 (2) (2013) 136–146. https://doi.org/10.1016/j.jsb.2013.09.023.

[10] R. Huang, M. Li, R.L. Gregory, Bacterial interactions in dental biofilm, Virulence 2 (5) (2011) 435–444. https://doi.org/10.4161/viru.2.5.16140.

[11] K. Lendenmann, J. Grogan, F.G. Oppenheim, Saliva and dental pellicle – a review, Adv. Dent. Res. 14 (2000) 22–28. https://doi.org/10.1177/08959374000140010301.

[12] C. Dawes, G.N. Jenkins, C.H. Tonge, The nomenclature of the integuments of the enamel surface of the teeth, Br. Dent. J. 115 (1963) 65–68.

[13] M. Hanning, The protective nature of the salivary pellicle, Int. Dental Journal 52 (S5) (2002) 417-423. https://doi.org/10.1111/j.1875-595X.2002.tb00731.x.

# [14] C.A. Haynes, W. Norde, Globular proteins at solid/liquid interfaces, Colloids Surf. B, 2 (6) (1994) 517-566. https://doi.org/10.1016/0927-7765(94)80066-9.

[15] B. Grohe, A. Taller, P.L. Vincent, L.D. Tieu, K.A. Rogers, A. Heiss, E.S. Sørensen, S. Mittler, H.A. Goldberg, G.K. Hunter, Crystallization of calcium oxalates is controlled by molecular hydrophilicity and specific polyanion-crystal interactions, Langmuir 25 (19) (2009) 11635–11646. https://doi.org/10.1021/la901145d.

[16] G. Goobes, R. Goobes, W.J. Shaw, J.M. Gibson, J.R. Long, V. Raghunathan, O. Schueler-Furman, J.M. Popham, D. Baker, C.T. Campbell, P.S. Stayton, G.P. Drobny, The structure, dynamics, and energetics of protein adsorption – lessons learned from adsorption of statherin to hydroxyapatite, Magn. Reson. Chem. 45 (2007) S32–S47. https://doi.org/10.1002/mrc.2123.

[17] V. Carniello, B.W. Peterson, H.C. van der Mei, H.J. Busscher, Physico-chemistry from initial bacterial adhesion to surface programmed biofilm growth, Adv. Colloid Interf. Science 261 (2018) 1–14. https://doi.org/10.1016/j.cis.2018.10.005.

[18] S. Shah, Statherin-role in biomimetic early caries management, Acta Scientific Dental Sciences 2 (6) (2018) 57-60.

[19] J.D.B. Featherstone, Dental caries: a dynamic disease process, Australian Dental Journal 53 (2008) 286–291. https://doi.org/10.1111/j.1834-7819.2008.00064.x.

[20] J.D.B. Featherstone, The science and practice of caries prevention, J. Am. Dent. Assoc. 131 (7) (2000) 887–899. https://doi.org/10.14219/jada.archive.2000.0307.

[21] J.D.B. Featherstone, Diffusion phenomena and enamel caries development. Cariology Today, International Congress Zűrich 1983, Karger, Basel,1984, pp. 259–268.

[22] N. Philip, State of the art enamel remineralization systems: the next frontier in caries management, Caries Res. 53 (2019) 284–295. https://doi.org/10.1159/000493031.

[23] Z.R. Zhou, J. Zheng, Oral Tribology, Proc. Instit. Mech. Eng. Part J 220 (8) (2006) 739-754. https://doi.org/10.1243/13506501JET145.

[24] N. Schlueter, T. Jaeggi, A. Lussi, Is dental erosion really a problem? Adv. Dental Res. 24 (2) (2012) 68-71. https://doi.org/10.1177/0022034512449836.

[25] S. Wongkhantee, V. Patanapiradej, C. Maneenut, D. Tantbirojn, Effect of acidic food and drinks on surface hardness of enamel, dentine, and tooth-coloured filling materials, J. Dentistry 34 (3) (2006) 214-220. https://doi.org/10.1016/j.jdent.2005.06.003.

[26] I. Azouzi, I. Kalghoum, D. Hadyaoui, B. Harzallah and M. Cherif, Principles and guidelines for managing tooth wear: a review, Int. Med. Care, 2 (1) (2018) 1-9. https://doi.org/10.15761/IMC.1000112.

[27] A.A. Algadhi, Tooth Surface Loss: Definitions, Prevention and Diagnosis, Saudi J. Oral Dent. Res., 6 (3) (2021) 129-133. https://doi.org/10.36348/sjodr.2021.v06i03.005.

[28] S.B. Mehta, S. Banerji, B.J. Millar, J.M. Suarez-Feito, Current concepts on the management of tooth wear: part 1. Assessment, treatment planning and strategies for the prevention and the passive management of tooth wear, Brit. Dental Journal 212 (1) (2012) 17-27. https://doi.org/10.1038/sj.bdj.2011.1099.

[29] J.A. Kaidonis, L.C. Richards, G.C. Townsend, Non-carious changes to tooth crowns, in: J.M. Graham, W.R. Hume (Eds.), Preservation and restoration of tooth structure, Knowledge Books and Software, Varsity Lakes Qld, Australia, 2005, pp. 47-60.

[30] J.O. Grippo, Abfractions: A new classification of hard tissue lesions of teeth, Journal of Esthetic and Restorative Dentistry, 3 (1) (1991) 14-19. https://doi.org/10.1111/j.1708-8240.1991.tb00799.x.

[31] Z.R. Zhou, J. Zheng, Tribology of dental materials: A review, J. Phys. D: Appl. Phys. 41 (11) (2008) 1-22. https://doi.org/10.1088/0022-3727/41/11/113001.

[32] A. Wiegand, A. Credé, C. Tschammler, T. Attin, T.T. Tauböck, Enamel wear by antagonistic restorative materials under erosive conditions, Clin. Oral Invest. 21 (9) (2017) 2689-2693. https://doi.org/10.1007/s00784-017-2071-9.

[33] T. Donovan, [C. Nguyen-Ngoc](https://onlinelibrary.wiley.com/action/doSearch?ContribAuthorStored=Nguyen-Ngoc%2C+Caroline), [I. Abd Alraheam](https://onlinelibrary.wiley.com/action/doSearch?ContribAuthorStored=Abd+Alraheam%2C+Islam), [K. Irusa](https://onlinelibrary.wiley.com/action/doSearch?ContribAuthorStored=Irusa%2C+Karina), Contemporary diagnosis and management of dental erosion, J. Esthet. Restor. Dent. 33 (1) (2021) 78–87. https://doi.org/10.1111/jerd.12706.

[34] D. Bartlett, C. Ganss, A. Lussi, Basic Erosive Wear Examination (BEWE): a new scoring system for scientific and clinical needs, Clin. Oral Investig. 12 (Suppl. 1) (2008) S65-68. https://doi.org/10.1007/s00784-007-0181-5.

[35] M. Goldberg, Prevention of enamel and dentin carious lesions, J.S.M. Dent. 8 (1) (2020) 1121.

[36] F. García-Godoy, M.J. Hicks, Maintaining the integrity of the enamel surface: the role of dental biofilm, saliva and preventive agents in enamel demineralization and remineralization, J. Am. Dent. Assoc. 139 (2008) Suppl: 25S-34S. https://doi.org/10.14219/jada.archive.2008.0352.

[37] W.L. Jongebloed, I. Molenaar and 1. Arends, Morphology and size-distribution of sound and acid-treated enamel crystallites, Calcif. Tissue Res. 19 (1975) 109–123. https://doi.org/10.1007/BF02563996.

[38] J.C. Voegel, R.M. Frank, High resolution electron microscopy of the human enamel apatite crystal and its carious dissolution, J. Biol. Buccale 2 (1) (1974) 39-50.

[39] K.A. DeRocher, P.J.M. Smeets, B.H. Goodge, M.J. Zachman, P.V. Balachandran, L. Stegbauer, M.J. Cohen, L.M. Gordon, J.M. Rondinelli, L.F. Kourkoutis, D. Joester, Chemical gradients in human enamel crystallites, Nature 583 (2020) 66–71. https://doi.org/10.1038/s41586-020-2433-3.

[40] C. Robinson, R.C. Shore, S.J. Brookes, S. Strafford, S.R. Wood, J. Kirkham, The chemistry of enamel caries, Crit. Rev. Oral. Biol. Med. 11(4) (2000) 481-495. https://doi.org/10.1177/10454411000110040601.

[41] St. Risnes, Ch. Li, On the method of revealing enamel structure by acid etching. Aspects of optimization and interpretation, Microsc. Res. Tech. 82 (2019) 1668–1680. https://doi.org/10.1002/jemt.23333.

[42] Z.-J. Cheng, X.-M. Wang, F.-Z. Cui, J. Ge, J.-X. Yan, 2009. The enamel softening and loss during early erosion studied by AFM, SEM and nanoindentation. Biomedical Materials 4, 015020. https://doi.org/10.1088/1748-6041/4/1/015020.

[43] W.L. Jongebloed, I. Molenaar, J. Arends, Effect of monofluorophosphate on the pathways followed by cariogenic agents, Caries Res. 9 (1975) 388-403. https://doi.org/10.1159/000260180.

[44] A. Van Nieuw Amerongen, J.G.M. Bolscher, E.C.I. Veerman, Salivary proteins: protective and diagnostic value in cariology?, Caries Res. 38(3) (2004) 247-253. https://doi.org/10.1159/000077762.

[45] L.A. Tabak, In defense of the oral cavity: the protective role of salivary secretions, Pediatr. Dent. 28(2) (2006) 110-117; discussion 192-198.

[46] B. Tomazic, M. Tomson and G.H. Nancollas, The growth of calcium phosphates on natural enamel, Calcif. Tissue Res. 19 (4) (1976) 263-271. https://doi.org/10.1007/BF02564009.

[47] E.C. Moreno, R.T. Zahradnik, A. Glazman and R. Hwu, Precipitation of hydroxyapatite from dilute solutions upon seeding, Calcif. Tissue Res. 24 (1) (1977) 47-57. https://doi.org/10.1007/BF02223296.

[48] G.H. Nancollas and B. Tomazic, Growth of calcium phosphate on hydroxyapatite crystals. Effect of supersaturation and ionic medium, J. Phys. Chem. 78 (22) (1974) 2218-2225. https://doi.org/10.1021/j100615a007.

[49] J.P. Barone and G.H. Nancollas, The seeded growth of calcium phosphates. The effect of solid/solution ratio in controlling the nature of the growth phase, J. Colloid Interface Sci. 62 (3) (1977) 421-431. https://doi.org/10.1016/0021-9797(77)90093-5.

[50] G.H. Nancollas, Enamel apatite nucleation and crystal growth, J. Dental Res. 58 (2 suppl.; spec. issue B) (1979) 861-870. https://doi.org/10.1177/00220345790580024001.

[51] B. Tomazic, M. Tomson, G.H. Nancollas, Growth of calcium phosphates on hydroxyapatite crystals: The effect of magnesium, Arch. Oral Biol. 20 (1975) 803-808.

[52] N.C. Blumenthal, F. Betts, A.S. Posner, Stabilization of amorphous calcium phosphate by Mg and ATP, Calc. Tis Res. 23 (1977) 245–250. https://doi.org/10.1007/BF02012793.

[53] M.T. Valente, E.B. Moffa, K.T.B. Crosara, Y.Z. Xiao, T.M. de Oliveira, M.A.D.M. Machado, W.L. Siqueira, Acquired enamel pellicle engineered peptides: effects on hydroxyapatite crystal growth, 2018. Scientific Reports 8, 3766. https://doi.org/10.1038/s41598-018-21854-4.

[54] J. Reyes-Gasga, E.F. Brès, High resolution STEM images of the human tooth enamel crystals, 2021. Appl. Sci. 11(16), 7477. https://doi.org/10.3390/app11167477.

[55] F. Yun, M.V. Swain, H. Chen, J. Cairney, J. Qu, G. Sha, H. Liu, S.P. Ringer, Y. Han, L. Liu, X. Zhang, R. Zheng, Nanoscale pathways for human tooth decay—Central planar defect, organic rich precipitate and high-angle grain boundary, 2020. Biomaterials 235, 119748. https://doi.org/10.1016/j.biomaterials.2019.119748.

[56] A.F. Marshall, K.R. Lawless, TEM study of the central dark line in enamel crystallites, J. Dent. Res. 60 (10) (1981) 1773–1782. https://doi.org/10.1177/00220345810600100801.

[57] E.F. Brès, J.C. Barry, J.L. Hutchison, A structural basis for the carious dissolution of the apatite crystals of human tooth enamel, Ultramicroscopy 12 (4) (1983) 367–372. https://doi.org/10.1016/0304-3991(83)90250-4.

[58] L.M. Gordon, M.J. Cohen, K.W. MacRenaris, J.D. Pasteris, T. Seda, D. Joester, Amorphous intergranular phases control the properties of rodent tooth enamel, Science 347 (6223) (2015) 746–750. https://doi.org/10.1126/science.1258950.
